# Enhancing the Phenolic Value of Polyphenol-Rich Extra Virgin Olive Oils Through Optimized Water Extraction, Macroporous Resin Recovery and Spray Drying

**DOI:** 10.3390/molecules31183298

**Published:** 2026-09-17

**Authors:** Athanasios Gerasopoulos, Kyriakos Kachrimanis, Diamanto Lazari

**Affiliations:** 1 Laboratory of Pharmacognosy, School of Pharmacy, Aristotle University of Thessaloniki, 54124 Thessaloniki, Greece; ageraso@pharm.auth.gr; 2 Department of Pharmaceutical Technology, School of Pharmacy, Aristotle University of Thessaloniki, 54124 Thessaloniki, Greece; kgk@pharm.auth.gr; 3 Center for Interdisciplinary Research & Innovation, Aristotle University of Thessaloniki, 54124 Thessaloniki, Greece

**Keywords:** phenolic compounds, water extraction, oleocanthal, oleacein, response surface methodology, DPPH, XAD resins

## Abstract

Olive oil polyphenols are well-established for their health-promoting properties. A recently emerged polyphenol-rich extra virgin olive oil (EVOO) was subjected to a water extraction process, optimized using response surface methodology (RSM). The effects of salt content and the water-to-oil ratio on the total phenolics were initially evaluated. Maximal phenolic yield was obtained by a water/EVOO ratio of 9.6:1 (*v*:*v*) and 0% added salt, although the addition of 3% salt provided an oil-free extract with a satisfactory phenolic yield compared to the optimized extract. Further, the phenolics of the optimized water extract were recovered using macroporous XAD resins. XAD-7HP resin and 75% ethanol/water eluent were selected based on comparatively improved adsorption/desorption and recovery data. Recovered phenolics were then encapsulated using spray drying and a maltodextrin carrier; the resulting powder was evaluated for physical and phenolic release properties. Phenolic composition was measured in all stages using ^1^H-NMR, revealing a phenolic yield of 39% in the extraction step, which was reduced through recovery and encapsulation to 25%; oleocanthal and oleacein were partitioned from olive oil to water extract by 55–50% while oleuropein and ligstroside aglycones only by 14.5–31.7%. The compositional pattern of water-extracted phenolics remained the same throughout recovery and encapsulation. These findings contribute to a moderately efficient, green procedure in developing innovative nutraceutical supplements/functional food ingredients.

## 1. Introduction

Extra virgin olive oil is known for its richness in unique polyphenols (such as oleocanthal, oleacein, ligstroside aglycone, oleuropein and hydroxytyrosol); olive oil consumption has been associated with lower all-cause mortality and improved lipid profiles when compared to saturated fats. Additionally, olive oil protects blood lipids from oxidative stress, thus contributing to general wellness [1]. The key health properties of EVOOs refer to their antioxidant [2], cardioprotective [3], and anti-inflammatory potential [4]; phenols like oleocanthal and oleacein reduce oxidative damage to LDL (bad cholesterol) and lower cardiovascular risk markers [5]. The bioactive compounds of olive oil act similarly to mild nonsteroidal anti-inflammatory drugs (NSAIDs) [4].

Approximately 90% of the total phenolics in olive oil polyphenols are classified mostly as secoiridoids (oleuropein and ligstroside in their aglycone forms, as well as their derivatives oleacein and oleocanthal). Olive oil also contains a minor fraction of phenolic compounds such as alcohols (hydroxytyrosol, tyrosol), flavonoids (luteolin and apigenin), lignans (i.e., (+)-pinoresinol), phenolic acids (i.e., p-coumaric, ferulic, cinnamic, caffeic, gallic, vanillic and syringic acid) and hydroxy-isocromans [6,7,8,9,10].

The European Food Safety Authority (EFSA) authorizes a strict health claim for oils containing at least 5 mg of hydroxytyrosol per 20 g of oil (EU health claim (Regulation (EU) No 432/2012)) [11].

Olive oil polyphenols are minor constituents of olive oil and can account for up to 2% of total weight [12,13]. Polyphenol content in the majority of retail EVOO ranges from under 10 mg/kg to over 530 mg/kg [14]. A literature survey indicated that the majority of olive oils produced across many countries and different conditions (variety, geography, growing conditions, fruit maturity) fall below or about the EFSA polyphenol threshold of 250 mg/kg even though they are all similarly labeled [6,7,8,9,10,15,16]. However, recently a trend toward “high” and “exceptionally high” has emerged in olive oil production; Diamantakos et al. [17] proposed that these terms correspond to phenolic contents of more than 500 mg/kg and more than 1200 mg/kg, respectively. Thus, over the current decade, as new insights and technological advances are introduced, research has shown increasing phenolic content levels in olive oils, ranging from 1000 mg/kg to ~4500 mg/kg [18,19,20,21,22,23].

Current research trends recognize the emerging opportunity of olive oils with “high” or “exceptionally high” phenolic content to expand their use beyond consumption as food; emphasis has been placed on the isolation/extraction of the bioactive olive oil phenolics due to their significant pharmacological properties.

For the extraction of the polyphenol fraction of olive oils, as an initial step, substantial research has been conducted using both conventional and unconventional solvents/methods; however, these studies have been performed mainly for analytical purposes. Thus, olive oil phenolics are usually extracted using the liquid–liquid or liquid-to-solid technique. The most popular liquid–liquid extraction procedures employ a variety of organic solvents and traditional methods of manual/mechanical agitation [22,24,25,26] or advanced techniques such as supercritical fluid extraction, ultrasound-assisted extraction and microwave-assisted extraction [27]. Currently, the IOOC-approved official technique of analysis for phenolics [24] advises preparing samples of phenolic compounds extracted from olive oil with 80% aqueous methanol (MeOH) (or a 4:1 MeOH-to-water ratio, *v*:*v*) and sonication. EVOO phenolics have been extracted using organic solvents, including ethanol [27], as well as natural deep eutectic (DES) systems studies [28,29,30,31], with water and methanol serving as controls for comparison purposes. Wani et al. [29] compared the subcritical treatment of olive oil using methanol and ethanol to water; in both studies using DES or subcritical conditions, the antioxidant activity decreased drastically for water extraction. The water capacity required to efficiently extract oleocanthal and EVOO phenolics as a self-emulsified nano-emulsion was also reported by Siddique et al. [32].

However, for the purpose of extracting phenolics from olive oil for later use as a supplement, these procedures can sometimes incur large energy costs and result in the generation of excessive solvent waste, which can be more dangerous to dispose of than the real agricultural waste itself. As a result, there is a need to develop “green” extraction methods. Among green solvents, water is a low-cost, non-hazardous polar extraction solvent. It has been demonstrated to efficiently extract a wide range of phenolic compounds with significant antioxidant activity from a variety of plants [18,19,20,21]. While the potential for extracting value-added bioactive polyphenols from olive leaves and olive fruit using water as an extractor/carrier has long been investigated, a very limited number of reports have been found for olive oil. Olive phenols are highly water soluble; therefore, they simply wash away in wastewater. The addition of water to olive oil extraction, particularly in malaxation and three-phase centrifuges, significantly reduces the oil’s phenolic content. Depending on the system used, more than 40% of phenols became waste [12]. In fact, no water addition boosted the oils’ secoiridoid content (+5–13%) [33], while water addition with olive leaf extract or wastewater during malaxation resulted in oils with a greater overall phenolic content than those obtained with water [34].

In a subsequent step, to recover olive oil phenolics from a water extract matrix, the use of macroporous resins was considered a green and efficient technology for any scale of production since the resins are relatively cheap and easy to operate and manage. Macroporous resins use non-covalent interactions (Van der Waals force, hydrogen bonding interaction, electrostatic force, complexation, and size sieving action) and physical properties to selectively separate target compounds from solutions. By varying the polarity and pore structure of the resin, these materials achieve highly specific compound isolation [35]. Polyaromatic Amberlite resins (XAD 4, XAD 7HP, and XAD 16N, among others) have been reported to concentrate and purify phenolics from olive mill wastewaters [36,37,38,39] and self-emulsified nano-emulsion from water/olive oil mixtures [32]. Further, Amberlite XAD 4 and XAD 16N are strongly hydrophobic, while Amberlite XAD-7HP is moderately polar/weakly hydrophobic [35,40,41,42,43].

As the final step, spray drying is a highly effective method used to convert liquid olive mill wastewater (OMWW) or olive leaf extracts into stable, concentrated and easily processable powders rich in biophenols. It has been employed to microencapsulate antioxidant compounds like hydroxytyrosol and oleuropein to protect them from heat and degradation [44,45,46,47].

The purpose of this study was to investigate and optimize a sustainable green extraction approach that uses an aqueous system to extract phenolics from polyphenol-rich extra virgin olive oils (EVOOs). EVOO with ~1000mg/kg of polyphenols was used to optimize extraction employing response surface methodology (RSM). The salt content (to minimize emulsification) and water-to-oil ratio were evaluated as extraction factors. Additional experiments were conducted to recover and purify the extract, comparing three Amberlite resins (XAD 4, XAD 7HP, and XAD 16N) and improving the operating conditions of polyphenol adsorption/desorption in order to identify the optimal resin and solvent. The most efficiently recovered extract was solidified by spray drying in the form of a dry powder, which was subsequently examined for its characteristics and phenolic release kinetics.

The total phenolic content (TPC) of the generated EVOO extract and recovered and encapsulated material was measured using the Folin–Ciocalteu technique, and the major phenolics were identified using ^1^H-NMR. This study aims to provide substantial insights into the process of manufacturing high-value-added products from high-phenolic EVOOs for the pharmaceutical and food industries via water extraction.

## 2. Results and Discussion

### 2.1. Optimization of Phenolic Extraction from Polyphenol-Rich Extra Virgin Olive Oils Using Water

As a first step, a sustainable water-based extraction process was optimized for the recovery of phenolics from a polyphenol-rich extra virgin olive oil (EVOO) using response surface methodology (RSM). A face-centered central composite design (FC-CCD) was applied to investigate the effects of the water-to-EVOO ratio and salt-content extraction parameters on the phenolic yield and radical scavenging activity (RSA) of the obtained extracts.

#### 2.1.1. Fitting the RSM Models

Extraction conditions were optimized through 14 randomized experimental runs to evaluate the effects of selected variables on TPC yield and their %RSA in high-phenolic EVOO. The experimental design (face-centered central composite design, FC-CCD), including the two independent variables and their corresponding coded and uncoded levels, is presented in Table 1. The extraction procedure was optimized using a second-order polynomial equation. The models were highly significant (*p* < 0.001) and well-fitted to the experimental TPC data (R^2^ value of 99.03), as well as to the %RSA (R^2^ value of 98.43), response (Table 2). Also, for both models, R^2^-adjusted and R^2^-predicted values were >90%, with a difference of less than 0.2 compared to R^2^, suggesting consistent accuracy and a significant correlation between observed and predicted data. Furthermore, lack-of-fit values were found to be non-significant (*p* > 0.05) for all produced models, indicating that the FC-CCD models accurately specified the relationship between the responses and the predictors (Table 2).

Multiple linear regression analysis was used to derive the regression coefficients for the dependent variables (Table 2), while ANOVA results are presented in Supplementary Table S1. A significant (*p* < 0.001) positive linear effect of % salt content (X1) was observed for both TPC and %RSA, indicating that increasing the salt content negatively affected the recovery of phenolic compounds and reduced the antioxidant activity of the EVOO water phenolic extracts. Similarly, for %RSA, a negative linear effect (*p* < 0.001) was noted for the water/EVOO ratio (X2) parameter. In contrast, a significant positive effect (*p* < 0.001) was observed regarding TPC response.

Furthermore, the quadratic effects of X_1_^2^ and X_2_^2^ on TPC and %RSA were significant (*p* < 0.001) and positive or negative, suggesting a nonlinear relationship between the examined extraction parameters and the selected responses. Moreover, the interaction effect X_1_X_2_ exhibited a significant negative effect (*p* < 0.001) for both TPC and %RSA. It should be noted that among the linear terms, the water/EVOO ratio parameter exhibited a greater influence on both TPC and %RSA responses than salt content, as indicated by its higher adjusted sum of squares (Supplementary Table S1).

The adequacy of the models was further assessed by plotting the predicted and experimental TPC values (Supplementary Figures S1 and S2). The models’ experimental and predicted responses, presented in Table 3, exhibit good correlation. The experimental and predicted responses exhibited strong correlations, with R^2^ values of 99.1% for TPC and 97.1% for %RSA, demonstrating the satisfactory predictive performance of the developed models. Lastly, the normal probability plots of the residuals revealed linear patterns, validating the normal distribution of errors and the model assumptions.

#### 2.1.2. Effect of Salt Content on Total Phenolic Content and %RSA

Salt was introduced in the experimental design as an emulsion breaker factor with the aim of producing oil-free water extracts, inducing phase separation and improving further handling. The selected salt range of 0–30% (*w*/*v*) was determined based on single-factor preliminary experiments, which showed that increasing the salt level resulted in a relatively satisfactory phenolic yield while promoting physical separation and a virtually “oil-free” water extract. The influence of salt content in the prepared water/EVOO mixtures on extract TPC yield and %RSA values was found to be statistically significant, as shown in the ANOVA (Supplementary Table S1).

**Figure 1:**
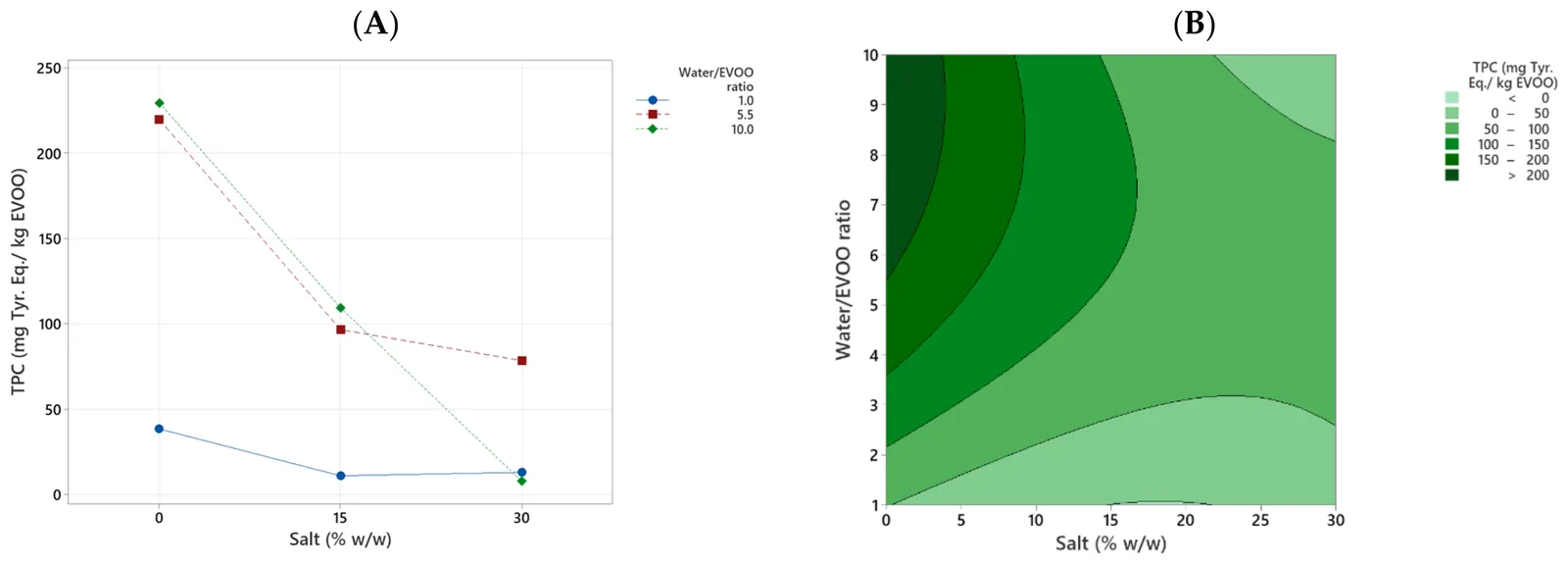
Main effects (**A**) and response surface/contour (**B**) plots showing the interactive effect of salt concentration and water/EVOO on total polyphenol content (TPC) (mg Tyr. Eq./kg EVOO).

Figure 1A depicts the main effects plot for the interaction between salt concentration percentage and water/EVOO ratio on the TPC of water extracts. The range of TPC for pure water (no salt added) samples was ~39–230 mg Tyr. Eq./kg EVOO (mean 160 mg Tyr Eq./kg EVOO), which was reduced by >50% as the concentration of salt in the water extract increased to 15% *w*/*v*. TPC was further significantly reduced when the salt concentration reached 30%.

**Figure 2:**
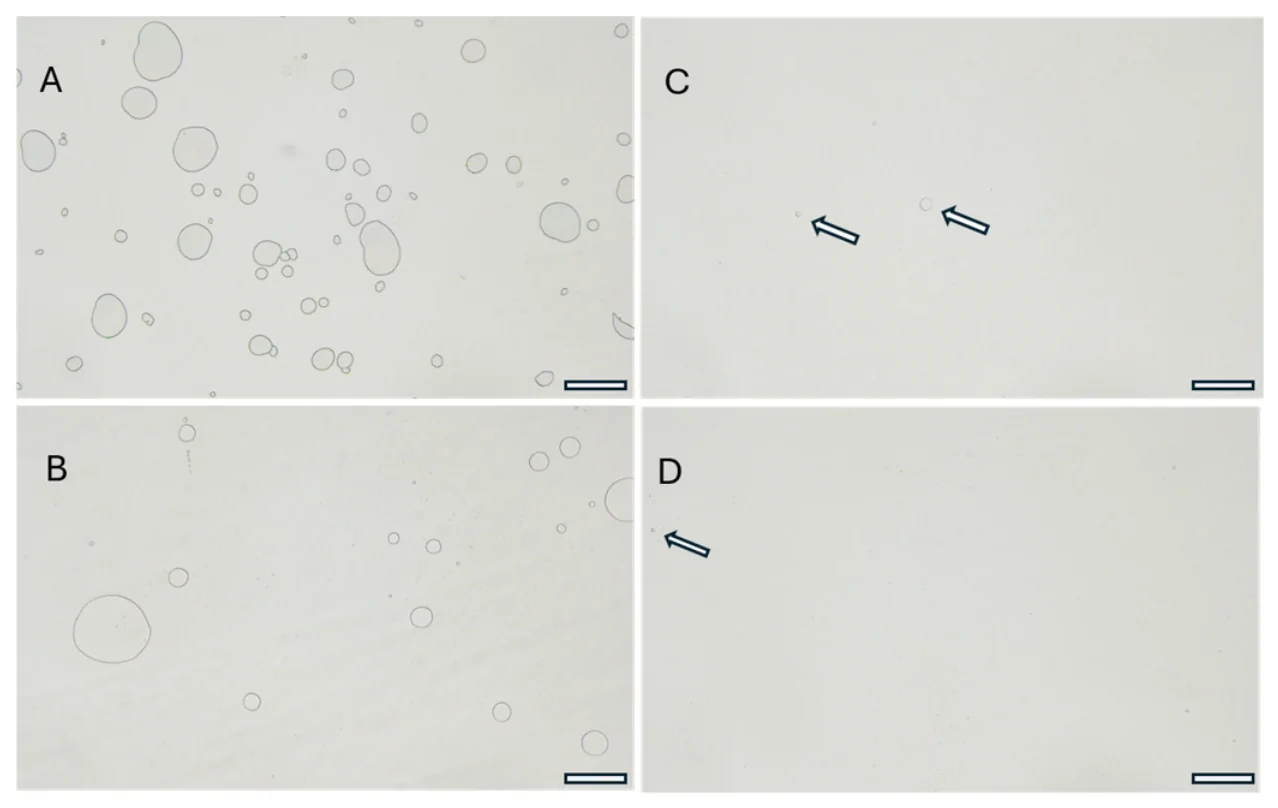
Microscope images of water extract containing 0 (pure water-(**A**)), 1 (**B**), 3 (**C**) and 5 (**D**) % salt. Arrows indicate oil droplets evident in the water extract. Scale bar: 100 µm.

The contour plot in Figure 1B revealed that when salt concentration increased from 0 to 15%, the TPC transferred from EVOO to water was largely determined by the water/EVOO ratio; within the 0–15% salt content range, the higher the ratio of water/EVOO employed, the higher the TPC yield. However, when salt was added at a higher concentration of 15–30%, TPC yield was significantly reduced, and this decrease was generally independent of the water/EVOO ratio. The highest TPC yield, within the 0–15% range, was obtained with a salt concentration of 0%, as shown in the main effects interaction plot in Figure 1A. Pure water, with no salt added, yielded a peak TPC value of 229.70 ± 0.17 at a water/EVOO ratio range of 5.5–10. However, examining this extract under an optical microscope revealed the presence of many oil droplets (Figure 2A). When water is mixed with olive oil, an emulsion is formed [32]. Adding 3% salt to water/EVOO mixtures resulted in a significantly improved oil-free water extract (Figure 2B–D) but only marginally affected TPC yield in the desirable EVOO/water ratio range (5.5–10). For this reason, these conditions were selected to produce a water extract to be utilized in downstream handling.

According to the literature, salt (NaCl) has been added at a concentration of 1–10% in the olive paste malaxation stage of the olive oil extraction process to influence hydrophobic–hydrophilic equilibria and enhance the coalescence phenomenon [48,49,50,51]. As a result, more than 80% of the oil phase separation was promoted along with polar and amphiphilic molecules [49,50]. Furthermore, due to the ability of salt to break down emulsions and the resulting alteration of the water-to-oil density ratio, higher extraction oil yields were recorded, while olive oil TPC [49,50,51] and quality were maintained [48].

Water-in-oil (W/O) emulsion systems, such as those made with olive oil, are primarily stabilized by steric hindrance (emulsifiers). The stability of W/O emulsions decreases with increasing salinity; when salt concentration increases, the W/O emulsion droplets become larger due to a quicker rate of aggregation and coalescence, reducing emulsion stability [52,53]. On the other hand, small amounts of salt (usually 0.7% to 1%) can significantly improve the stability of a W/O emulsion [54] while increasing lipid oxidation [55].

The decreased yield of phenolic compounds due to salt addition could be related to the phenolic profile of the EVOO, with EVOO polyphenols exhibiting a variety of polarity and partition characteristics in the water/EVOO or salt/water/EVOO system. Similar results were reported by Laurenti et al. [56], who examined the partition of olive oil phenolics to the water phase depending on the presence of various salts (including NaCl) during the milling process. More specifically, the authors highlight that a decrease in the amount of polyphenols (i.e., oleocanthal, oleacein) transferred to the aqueous phase was observed, which was proportional to the increase in salt concentration up to its saturation limit. Despite the partial transfer of phenolics to the aqueous phase in terms of initial and residual EVOO quality parameters, acidity and peroxide value remained relatively constant. However, a slight decrease in K values and organoleptic parameters was seen as a result of phenolic removal by water (Supplementary Table S2).

**Figure 3:**
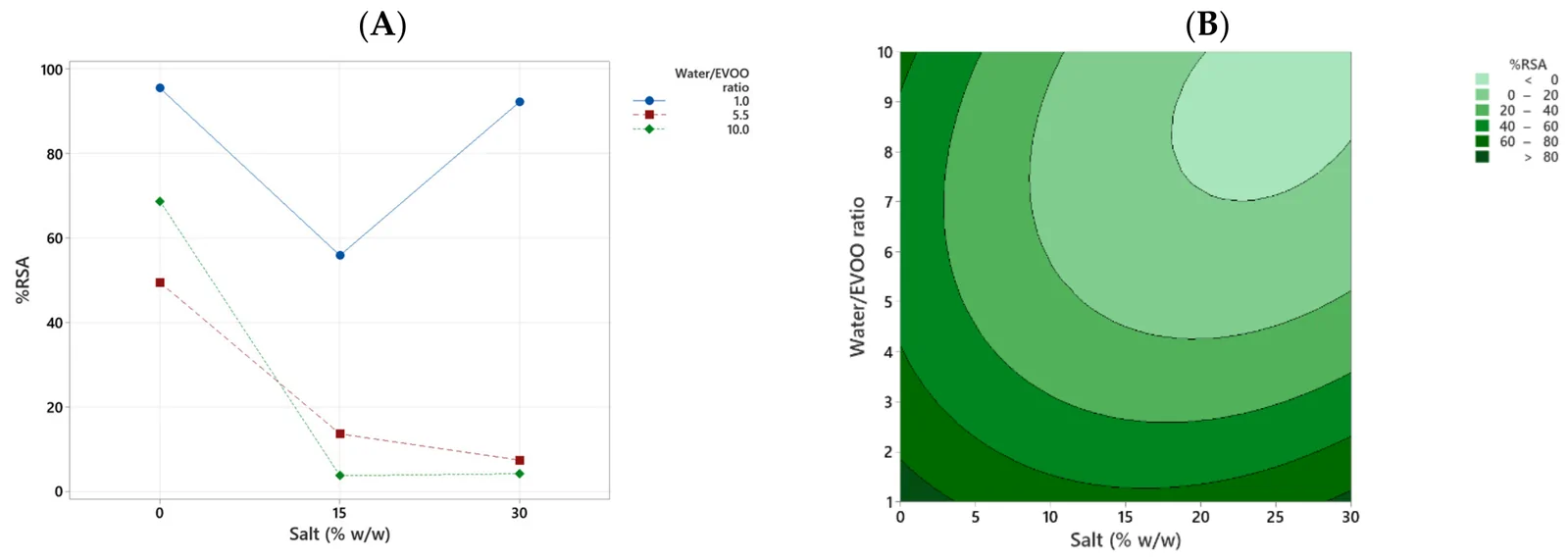
Main effects (**A**) and response surface/contour (**B**) plots showing the interactive effect of salt concentration and water/EVOO ratio on the percentage of radical scavenging inhibition (%RSA).

Moreover, Figure 3A shows the influence of the salt content in the prepared water/EVOO mixtures on extract RSA. The range of pure water (no salt added) was 50–96% (mean was 72%). However, the pattern of %RSA, as affected by the interaction of salt with the water/EVOO ratio, appeared to be the opposite of that of TPC. Maximum %RSA was obtained at 1–3% salt content levels and also at very low water/EVOO ratio values (1–2). The %RSA exhibited an initial reduction as salt increased to 15%, then increased again as salt increased to 30%, but remained lower than that observed at the 0% salt level; when the ratio of water/EVOO was increased to 10%, RSA was reduced to as little as 5–10% (at 30% salt content) (Figure 3B).

Although it seemed that the pattern of extraction of %RSA was opposite to that of TPC, this may be explained by the principles of mass transfer, since TPC was expressed in mg Tyr Eq. kg of EVOO basis; the extract produced under a low water/EVOO ratio (e.g., value of 1, or 1 kg of water mixed with 1 kg of EVOO) had more phenolics and thus greater %RSA compared to the higher ones prepared by applying increased water/EVOO ratio values (e.g., for water/EVOO ratio value of 10, or 10 kg of water mixed with 1 kg of EVOO). Mass transfer indicates that the concentration gradient acts as a driving force between EVOO and the water (solvent); mass transfer is better when a lower water/EVOO ratio is used [33,57,58]. Another factor might be related to the hydrophilicity of the phenolic bioactives; polar phenolic compounds (e.g., hydroxytyrosol and tyrosol) have a strong affinity for water, causing them to migrate out of the oil when water is introduced [59]. When the water approaches its maximum solubility limit for the extracted phenolic compounds, the gradient diminishes, and mass transfer also diminishes and eventually comes to a complete halt [60,61]. Similar results for the extraction of phenolic compounds have been reported when solvent-to-solid ratio systems were used [62,63].

The above data are also reflected in the very poor correlation of TPC and %RSA noted (Supplementary Figure S3). It should be mentioned that the DPPH assay and TPC (measured by Folin–Ciocalteu assay) values do not always correlate, as reported in the literature, i.e., for secoiridoids [64,65,66,67,68]. The DPPH assay is heavily influenced by the specific antioxidant structure–activity relationship [68]. For example, hydroxytyrosol-type structures act as strong hydrogen donors, whereas tyrosol-type structures bearing a single para-hydroxyl exhibit comparatively weaker radical scavenging activity [69], although both share a similar Folin–Ciocalteu assay.

#### 2.1.3. Effect of Water/EVOO Ratio on Total Phenolic Content and %RSA

The water/EVOO ratio is considered an important extraction factor since it controls the partitioning of bioactive compounds from the oil matrix into the water phase. The selected range (0–10) was determined based on single-factor preliminary experiments, in which applying a ratio above 10 did not result in enhanced phenolic yield. According to the ANOVA shown in Supplementary Table S1, the water/EVOO ratio factor significantly affected TPC yield.

Interaction and contour plots (Figure 1) show the effects of water/EVOO ratio and salt content on phenolic yield; phenolic yield increased as the ratio’s value increased. Such an interaction is common in extraction techniques. By effectively penetrating the targeted matrix, a higher ratio typically enables the extractant (water) to actively interact and solubilize the bioactives [64,70,71,72]. In principle, the ratio should be as low as feasible primarily for handling purposes; however, using a far greater ratio in certain cases may result in a lower yield. This is presumably because the extraction medium is oversaturated, a condition that limits mass transfer rates [72,73,74,75]. The application of large amounts of water may bring potential emulsification, which can in turn hamper physical phase separation. Nevertheless, upon centrifugation, all water/EVOO mixtures—across all tested ratios—achieved typical phase separation. Additionally, a higher water/EVOO ratio could impose a practical restriction on later processing steps, such as the recovery of bioactives; when freeze-drying is employed, more energy to evaporate the excess water would be needed, and when macroporous resins are used, significant handling and time would be required.

A ratio of 5.5–10:1 was sufficient to obtain the highest yield; solubilization of bioactives within the oil matrix may be hampered by EVOO with a higher or lower phenolic content and/or with a different compositional profile. While complex secoiridoids might be broken down into more hydrophilic simple phenolics as a result of hydrolytic reactions, or subjected to losses due to oxidation during manufacturing and storage, olive oil would contain polyphenol molecules of a wide polarity range [76,77].

Olive oil’s phenolic compounds, including oleocanthal, oleacein, and oleuropein, are primarily responsible for the oil’s potent antioxidant and anti-inflammatory properties. Unlike smaller, more hydrophilic phenols (like hydroxytyrosol and tyrosol), these larger, lipophilic compounds dictate the distinctive bitter taste and throat-stinging pungency of high-quality, fresh EVOO [78]. This is supported by the determined partition coefficients for EVOO polyphenols [79]. Compounds like oleocanthal and oleacein feature complex ester structures and a diformyl core. Their non-polar (hydrophobic) characteristics give them a lower partition coefficient in water, causing them to readily dissolve and remain retained within the lipid phase of the olive [80]. On the other hand, compounds like hydroxytyrosol and tyrosol are much smaller, simpler molecules. Their hydroxyl groups make them highly polar, resulting in higher solubility in water than in oil. Thus, during olive oil extraction, a large majority of these compounds are lost to the olive mill wastewater and pomace. These key differences in solubility stem from the unique chemical structures of these compounds, which dictate how they distribute themselves between the lipid (oil) and aqueous (water) phases during olive oil extraction or during extraction from the olive oil using an extractant.

TPC data shown in Figure 1 indicate that the maximum yield of 229.70 ± 0.17 mg Tyr Eq./kg EVOO was obtained when a water/EVOO ratio of 10:1 was used (Table 3). According to the data presented in Table 4, the extraction yield obtained was ~39%; although it can be characterized as moderate, it simply allows the use of EVOO after its extraction depending on the initial TPC. An extraction yield of ~50% was reported on the side of the extraction of olive oil phenolics using various deep eutectic solvents (DES) [28]. Also, in studying the extraction potential of DES [81,82] for phenolics from olive pomace and comparing DES with the water control, they concluded that DES extracted 7-fold more phenolic compounds than water. The comparative results of Chanioti and Tzia [82] for ethanol or water extractants were similar. In general, water is a less effective extractant compared to organic solvents or to DES [83].

Figure 3 shows the main effects of water/EVOO ratio on %RSA. The influence of the water (as a solvent)-to-EVOO ratio was studied by extracting the same quantities of EVOO samples using different quantities of water. Various water/EVOO ratios were tested, with values ranging from 1 to 10 (*v*:*v*). It can be observed that the %RSA was strongly affected by the water/EVOO ratio, with a *p*-value < 0.001 (Supplementary Table S1). The extraction of bioactive compounds from the EVOO matrix by water was related to their ratio (water/EVOO). The experimental data indicated that low water/EVOO ratios (ranging from 1 to 5.5) resulted in increased %RSA compared to the higher ones’ (ranging from 5.5 to 10). This is attributed to the dilution effect, as discussed earlier.

#### 2.1.4. Optimization of Total Phenolic Content and %RSA

The optimal extraction conditions were obtained by maximizing the TPC response and are shown in Table 4. Further experimentation utilizing points inside the examined RSM area was conducted to ensure the accuracy and predictive reliability of the derived model (Supplementary Table S3). The use of water without added salt and a water/EVOO ratio of 9.6:1 is proposed, yielding a TPC value of 252.12 ± 0.384 mg Tyr. Eq./kg EVOO. As observed in Table 4, the strong agreement between the predicted optimum and the experimental results confirms the validity of the proposed model. It should be noted that the produced extract was also evaluated in terms of its %RSA through the DPPH assay, yielding a relatively high %RSA value of 70.06 ± 0.60.

The optimal extracts’ phenolic profiles were determined using HPLC-DAD and ^1^H-NMR analysis and supported the presence of elevated concentrations of oleocanthal across all EVOOs.

#### 2.1.5. Folin–Ciocalteu, HPLC-DAD and ^1^H-NMR Analysis of Phenolic Compound Profiles for EVOO and Water Extract

The phenolic profile of EVOO was determined using HPLC-DAD analysis according to the official IOC method [24] (Supplementary Figure S4 and Table S4). Furthermore, the TPC and phenolic profiles of EVOO and the water extract produced under optimum conditions for the water/EVOO ratio and 3% salt content (Table 5, Supplementary Table S5) were determined using ^1^H-NMR [84] (Table 5 and Supplementary Figure S5).

Olive oil contains a minor fraction of phenolic compounds such as alcohols (hydroxytyrosol, tyrosol), flavonoids (luteolin and apigenin), lignans (i.e., (+)-pinoresinol), phenolic acids (i.e., p-coumaric, ferulic, cinnamic, caffeic, gallic, vanillic and syringic acid) and hydroxy-isocromans [6,7,8,9,10]. The minor fraction of phenolic compounds is shown in Supplementary Table S5; since the level of this fraction was very low (<10%) compared to primary secoiridoid fraction (oleocanthal, oleacein, oleuropein and ligstroside) [85] only the latter were measured after water extraction, XAD-7HP recovery and encapsulation.

The TPC measured by Folin–Ciocalteu and ^1^H-NMR analysis of the polyphenolic EVOO used in this study was 728.51 and 1050.92 mg/kg, respectively. The difference in TPC measured by the two methods was expected and has been documented in the literature [86].

Water extracts produced under the optimum conditions yielded 276.50 and 410.41 mg/kg according to Folin–Ciocalteu and ^1^H-NMR analysis methods, respectively; the corresponding percentages of phenolics transferred from EVOO to water extract were 37.95 and 39.05%, respectively.

Oleocanthal and oleacein were among the most abundant compounds present in the olive oil [30,32]. Oleocanthal was present at 297.43 ± 3.34 mg/kg in EVOO and 160.40 ± 3.35 mg/kg in the water extract. Oleacein, present at levels similar to those of oleocanthal, reached concentrations of 274.40 ± 7.03 and 137.20 ± 3.51 mg/kg in EVOO and the water extract, respectively. These values fall within the concentration ranges reported in the literature [34,84,87,88,89].

However, the oleuropein and ligstroside aglycone contents of the EVOO were 228.47 ± 4.15 and 250.61 ± 3.97, respectively; these levels are in agreement with the ranges [17,84,90] reported in the literature for high-phenolic EVOOs; of those values, only 33.23 ± 5.18-and 79.56 ± 4.18 mg/kg of the oleuropein and ligstroside aglycones, respectively, were transferred to the water extract.

Garcia et al. [28], in studying the extraction of olive oil with DES, used water and 80% methanol/water mixtures as controls and also reported that oleocanthal and oleacein were among the most abundant compounds in their olive oil samples and that the percentage of phenolic compounds extracted by water was about 50%; of those amounts, most of oleacein (~73%) was transferred to water, while oleocanthal showed the least transfer (~17%). Even though oleuropein and ligstroside aglycone contents were marginal (by <6-fold) compared to those of oleocanthal and oleacein, only 50% were transferred to water.

Siddique et al. [32], using water to produce emulsified extracts, reported oleocanthal yields from different olive oil sources ranging from 67.13 to 98.86%, as measured by quantitative ^1^H-NMR.

Extra virgin olive oil (EVOO) phenolics, such as oleocanthal and oleuropein aglycone, dissolve in both polar and non-polar environments, making them amphiphilic and allowing them to partition across oil–water interfaces [32,79,91]. Oleocanthal and oleacein have been reported to undergo a spontaneous interaction with H_2_O, forming a more polar, water-soluble acetal monohydrate through the addition of water to the C-3 aldehyde [84]. It appears that oleuropein and ligstroside aglycones were partitioned to water in lower percentages compared to their dialdehyde derivatives (oleocanthal and oleacein), probably partly due to their inability to form the more polar, water-soluble hydrated forms, as was the case with oleocanthal and oleacein [84].

Significant differences in TPC experimental data between the colorimetric Folin–Ciocalteu method and the HPLC-DAD and ^1^H-NMR methods were noted, with the former revealing values up to two times lower. This could be attributable to the higher selectivity of the HPLC-DAD and ^1^H-NMR methods compared to the Folin–Ciocalteu method, as well as to the fact that the Folin–Ciocalteu method measures in reference equivalents. Olmo-García et al. [92] and Ricciutelli et al. [93] found similar conclusions after thoroughly examining the identified differences between the Folin–Ciocalteu and HPLC-DAD methods. The slight discrepancies observed between HPLC-DAD and ^1^H-NMR in the present study are consistent with previous reports in the literature, where differences between the two analytical techniques have also been documented. However, the close agreement between the two methods indicates that the observed differences are attributable to their different quantification principles. HPLC-DAD quantification may be influenced by the limited availability of authentic standards and the different UV response factors of individual secoiridoids, whereas quantitative ^1^H-NMR provides a direct measurement based on signal integrals that are proportional to the number of resonating nuclei [94,95]. In support of this incentive, an interlaboratory harmonization study by Tsimidou et al. [96], which compared five liquid-chromatographic protocols (four HPLC/UHPLC-DAD variants and one LC-HRMS) with quantitative ^1^H-NMR across five expert laboratories, reported substantial differences in the absolute values obtained for identical samples; however, the methods remained statistically related. Although a strong correlation among the methods in terms of total phenolics is evident (in our study as well), this is not indicative of the discrepancies found in absolute values for the individual compounds. The observed differences could be attributed to, i.e., the inherent limitations of chromatographic methods for secoiridoid quantification that have been repeatedly documented, in particular the complexity and heterogeneity of the EVOO phenolic profile (co-eluting derivatives, diastereomers and isomers) and the formation of artifacts when protic solvents are used in sample preparation or as mobile phases. For this particular case, Celano et al. [97] demonstrated that oleocanthal and oleacein, which are open dialdehydes, react with methanol and water to form monohydrate, methyl-hemiacetal and dimethyl-acetal derivatives; in aqueous methanol—the solvent used for the HPLC-DAD analysis—these compounds could generate additional, partly co-eluting peaks that complicate integration and reduce the accuracy and reproducibility of the measurement. On the other hand, the NMR technique, although rapid and reliable, possesses the limitations of instrumental sensitivity, sample instability in CDCl_3_ solution and lack of quantification of the plethora of secoiridoids present in olive oil [94].

Considering the nutraceutical potential of EVOO phenolics and their recent evaluation in clinical studies, including metabolic syndrome parameters (OleoMetS) [98], early-stage chronic lymphocytic leukemia [99], mild cognitive impairment (MICOIL Pilot Study) [100], and oxidative and inflammatory biomarkers (OLIVAUS) [101], a reliable analytical method of quantification of the individual phenolic compounds is essential for their commercialization as supplements, medicinal formulations, or food ingredients.

### 2.2. Recovery of Phenolic Compounds Using XAD Macroporous Resins

Natural extracts (e.g., originating from plant biomass or agro-industrial wastewaters) are often passed through an XAD-packed column because, in the literature, they contain a mixture of components, including different classes of phenolics such as phenolic acids and flavonoids, for purification reasons. Because each phenolic compound has a unique chemical structure (e.g., varying numbers of hydroxyl groups, aromatic moieties), they interact with macroporous resins at different affinities, i.e., higher molecular weight phenols may not be able to access all the available adsorption surface inside the pore structure of the resin, and if one adsorbed molecule occupies one adsorption site, higher molecular weight phenols will result in higher values of adsorbed mass for a fixed number of adsorption sites [102]; the interaction of phenolic molecules and resin structures combines multiple mechanisms—physical forces and hydrogen bonding (can adsorb phenolic acids via π–π interactions), which depend on the polarity and surface area of the resin—resulting in complex, compound-specific adsorption and desorption behaviors [41,102] and sorbate-sorbent and solvent-associated interactions [103].

On this basis, three XAD resins, namely XAD-4, XAD-7HP, and XAD-16N, with proper adsorption conditions, were chosen to evaluate the adsorption behavior of the phenolics from the water extract. For the XAD experiment, a water extract with a water-to-EVOO ratio of 9.6 and a salt content of 3% was used since it is virtually oil-free yet still provides a satisfying phenolic yield compared to the optimized extract, as stated earlier (Section 2.1.2).

The adsorption and desorption characteristics of total polyphenols on the three resins are shown in Table 6. For the selection of the resin that provides better adsorption/desorption performance, static (batch) experiments were conducted. XAD-7HP exhibited the strongest adsorption capacity (4.04 mg/g dry resin), followed by XAD-16N and XAD-4 (3.9 and 3.2 mg/g dry resin, respectively). XAD-7HP also presented the highest adsorption rate (97.39%) of the phenolics present in the aqueous EVOO extract, compared with XAD-16N and XAD-4 (with adsorption rates of 94.08% and 77.55%, respectively). Furthermore, XAD-7HP, compared with the XAD-4 and XAD-16N resins, showed the highest desorption capacity (2.690 mg/g dry resin).

The superior adsorption performance of XAD-7HP may be related to the relatively polar nature of the polymethacrylic resin, whose carbonyl groups may participate in the formation of hydrogen bonds with the functional groups (hydroxyl moieties) of the phenolic compounds. In contrast, XAD-16N and XAD-4 are polystyrene-divinylbenzene (PS/DVB)-based resins and are considered hydrophobic. Combined with the increased polarity of the examined aqueous extract of EVOO polyphenols, lower adsorption capacity is expected. However, the adsorption behavior of phenolics on the resins depends on various other factors such as resin polarity, pore structure, surface area and the particular chemical characteristics of the individual phenolic compounds.

In fuller detail, phenolic profile analyses of the optimized aqueous EVOO phenolic extract (Section 2.1.5) revealed that it consisted predominantly of oleocanthal, oleacein, oleuropein aglycone, and ligstroside aglycone (Table 5). Also, unidentified oleuropein and ligstroside derivatives, as well as known phenolic acids and other minor compounds, were also observed, constituting less than 10% of the total phenolics of the extract. The primary phenolics, oleocanthal and oleacein, are reported as relatively hydrophobic yet water soluble, exhibiting logP values of 1.47 and 1.11, respectively [104]. It should be noted that the compounds mentioned can spontaneously react with water to form mixtures of hemiacetals and acetals, which may contribute to an increase in their polarity [105]. Considering the dipole moments of XAD-4, XAD-7HP, and XAD-16N (0.3, 1.8 and 0.8, respectively), the higher adsorption capacity of XAD-7HP could therefore be attributed to its greater polarity and therefore increased affinity with the examined phenolics. The higher adsorption capacity of XAD-16N than XAD-4 is not to be neglected, since it highlights that additional differences in the surface area and pore diameter can have a considerable impact on phenolic penetration, interactions with resin particle surfaces, and, finally, retention. For instance, large pores are reported to increase the accessibility of phenolics of high molecular weight, such as procyanidin B2, to adsorption sites, while for lower molecular weight phenolics, i.e., gallic acid and epigallocatechin, smaller pores trap them more easily, enhancing their retention [106].

Multiple mechanisms describe the adsorption of phenolic compounds onto XAD resins. Pan et al. [107] suggested that hydrogen bonds play a vital role in the adsorption of phenolic compounds onto XAD-4 and XAD-7 resins, along with hydrophobic interactions. In parallel, π–π conjugation between the aromatic moieties of the phenolics and the benzene rings of the resins is proposed as a mechanism of adsorption [108]. Thus, the position and nature of substituents on the aromatic ring of phenolics could influence the electron density and π-electron delocalization, potentially affecting their interactions with the resin. Pompeu et al. [109], investigating the adsorption mechanisms of different classes of phenolic compounds on macroporous resins, including XAD-7 and XAD-16, proposed that indeed the π–π stacking interactions between the aromatic rings of the adsorbates and the delocalized π-electron systems of the resin contribute significantly to the adsorption process.

Finally, the present findings regarding the adsorption and desorption process are consistent with a study by Sahin et al. [110], where the adsorption/desorption of oleuropein on XAD-2, XAD-4, XAD-7HP, and XAD-16 was examined. The results indicated the superior performance of XAD-7HP, followed by XAD-16. Furthermore, Johnson et al. [111] confirmed the increased affinity of the XAD resins studied with the EVOO phenolics, observing, however, that XAD-7HP exhibited lower adsorption capacity only for hydroxytyrosol and tyrosol, compounds that in our EVOO phenolic profile are of minor interest. In contrast, excellent performance was found for oleocanthal, oleacein, oleuropein and ligstroside aglycone. Overall, the present results support the suitability of XAD-7HP, followed by XAD-16N, for the adsorption and recovery of the phenolic compounds present in the aqueous EVOO extract.

**Figure 4:**
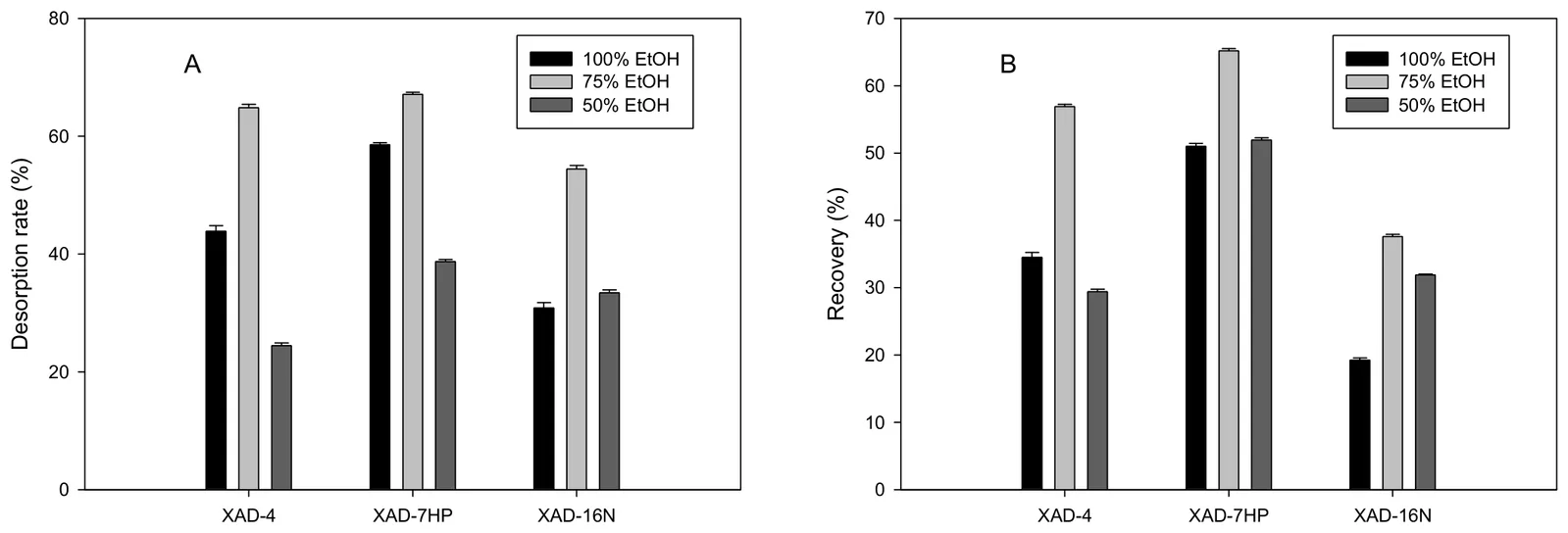
Desorption rate (**A**) and recovery rate (**B**) of the olive oil water extract phenolics for three resins eluted with different ethanol/water mixtures.

In the static desorption tests, 100, 75 and 50% ethanol were also employed. According to Figure 4A, when 75% ethanol was used as a desorption solvent, the highest desorption rate was observed for all resins examined. Desorption rates followed the same pattern with respect to desorption solvent (ethanol/water mixtures). As shown in Figure 4B, the recovery rates of olive oil phenolic with ethanol for these resins were significantly different; XAD-7HP exhibited the highest recovery (65.2%), followed by XAD-16N and XAD-4 (56.8 and 51.0%, respectively). Because XAD-7HP was more effective than the other resins when using a 75% ethanol solution as eluent, it was selected for additional research. Static tests for the recovery and concentration of EVOO extract polyphenols showed that optimal conditions, combining a green perspective with good adsorption–desorption yield, were those using XAD-7HP resin.

Amberlite resin properties have been reported to affect the adsorption and desorption capacity of olive wastewater phenolics [35]; in this study, results indicated that XAD-7HP resin with moderate polarity and a low surface area showed better adsorption capacity.

**Figure 5:**
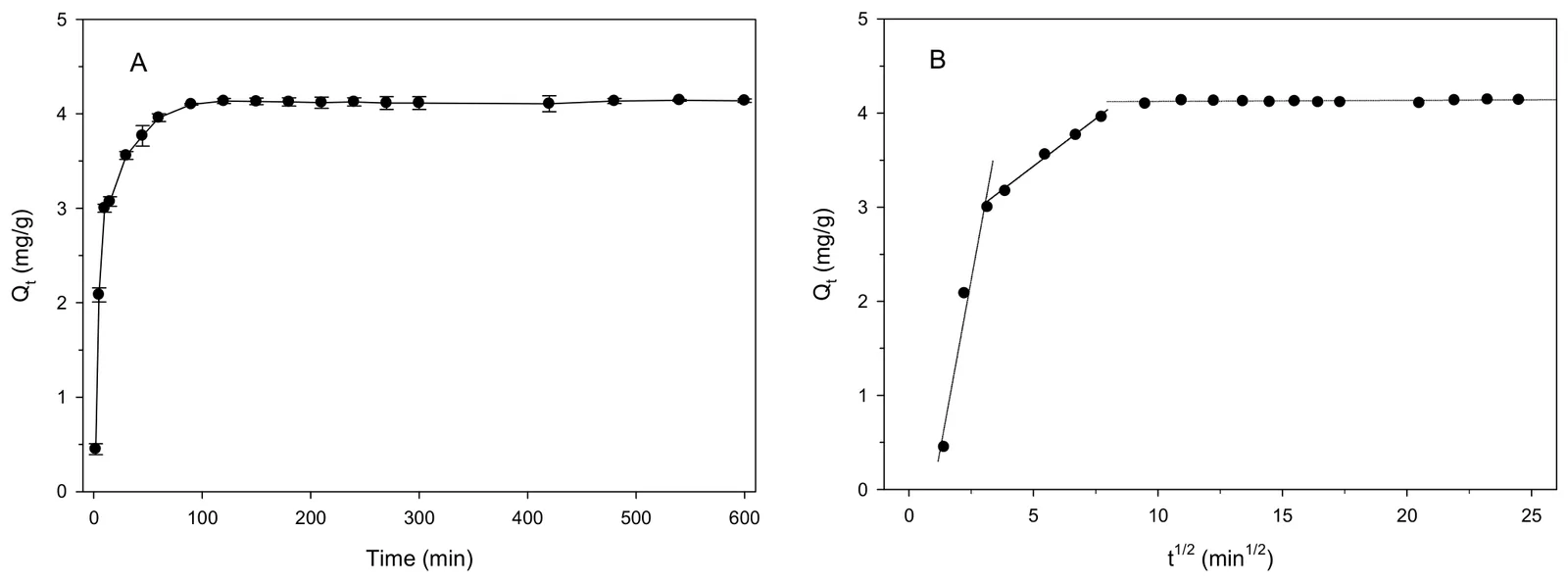
Adsorption (**A**) and diffusion (**B**) kinetics of phenolics for XAD-7HP.

The XAD-7HP adsorption time curves are given in Figure 5. The kinetic model fitting equations are shown in Table 7. Kinetic models have been used to investigate the mechanism of adsorption and possible rate-controlling steps. The adsorption capacity went through three stages: a fast rise between 0 and 15 min, followed by a slower increase to 60 min and an equilibrium stage that was reached after 120 min. The results revealed that the XAD-7HP resin exhibited rapid adsorption throughout the whole process. In order to further illuminate the adsorption characteristics of the resins used, three kinds of classical kinetic models were employed. The pseudo-first-order model, usually describing the initial, rapid stage of adsorption, indicated that unoccupied active sites on the XAD-7HP resin are highly abundant. The pseudo-second-order model, describing the whole adsorption process across the entire time range, indicated that the rate-limiting step throughout the entire process is chemisorption [112]. It can be observed that the pseudo-second-order model fitted better (R^2^ = 0.9997) to the adsorption data for XAD-7HP; this indicated that the adsorption process is controlled by the interaction of polyphenols and the active sites of the resin available, or by both the polyphenol concentration and resin surface area as factors [109]. This suggests that the adsorption process is limited by chemisorption, involving exchange or sharing of electrons between the resins and functional groups of EVOO phenolics [35,102,109,113,114,115].

Moreover, the entire process consisted of three stages, usually including boundary layer diffusion, the gradual adsorption stage and the final equilibrium stage [35,109]. This is supported by the intraparticle diffusion model, which showed a relatively good fit to the experimental data (R^2^ = 0.9644). However, the positive intercept ($C_{i}$ > 0) indicates that intraparticle diffusion was not the sole rate-controlling step, suggesting that other mechanisms, such as external film diffusion, also contributed to the mass transfer/adsorption process [114].

#### Folin–Ciocalteu and ^1^H-NMR Analysis of Recovered Phenolic Compounds

To remove the water solvent, extracts were passed through a column filled with XAD-7HP resin, and the Folin–Ciocalteu test in the eluent water confirmed the retention of phenolics by the XAD-7HP resin with a minimal loss of 1.5%. Following this, the adsorbed phenols were eluted by 75% ethanol as described in the previous section. As seen in Table 5, the amounts of the major secoiridoids determined were further slightly reduced; however, a similar pattern was observed for all individual phenolics. The percent recovery of water-extracted phenols through XAD-7HP resin was as high as 92.8%. This loss was probably limited by prior adsorption and desorption resin optimization; monitoring of phenolics in ethanol eluent through eight consecutive bed volumes ensured increased phenolic recovery. Alternatively, the loss was attributed to either degradation of phenolic molecules or the inability of the resin to adsorb them (Table 5).

The recovery rate of phenolic compounds from olive processing waste using Amberlite XAD resins generally ranges from 76% to 82% during the desorption/recovery phase, following an adsorption efficiency that can capture a major portion of the polyphenols. For XAD-7HP resin, the percentage recovery of total phenols varies based on the specific desorption solvent and wastewater source, generally achieving high desorption and mobilization ratios of up to 90% to 95% when optimal eluents like ethanol are used [116,117,118].

### 2.3. Encapsulation of Phenolic Compounds with Spray Drying and Powder Characterization

The encapsulation experiments were conducted following the elution of phenolics retained in the XAD-7HP resin, their concentration and reconstitution utilizing maltodextrin (MD) as a wall material. The spray-drying conditions were chosen based on literature reports of spray drying of phenolic extracts [45]. The product recovery, encapsulation efficiency, and basic physicochemical properties of the produced powder were determined, including particle size distribution, flowability properties, moisture, hygroscopicity, color parameters, total phenolic content, and the concentrations of the major individual phenolics present in the final product. In addition, the morphology of the particles was examined in order to more fully assess the quality, stability and technological suitability of the encapsulated product.

Regarding the product recovery, this was exceptionally high and formed at 84.1%, supporting that the spray-drying parameters, as well as the wall material chosen, were suitable for the process. This value falls within the range reported in the literature for phytochemical extracts subjected to spray drying using MD as a wall material [44,119,120,121].

At the same time, the total phenolic content, which exceeded 2 mg/g d.w. (Table 5), further indicates that the encapsulated product retained a considerable phenolic load following spray drying, which is linked to its potential functional and antioxidant value. This was further supported by the ^1^H-NMR phenolic analysis (Table 5), which confirmed that no substantial loss of the major individual phenolic compounds was observed.

Oleuropein aglycone exhibited differential retention, rising from 33.88 mg/kg in the extract after desorption to 390.47 mg/kg in spray-dried powder, compared with oleocanthal and oleacein. The observed differences reflect the fraction of each compound that was retained in the final encapsulated product. Focusing on the wall material utilized, MD functions as a protective matrix, forming a film or barrier that shields the entrapped phenolics from external factors [122] by rapid skin formation during atomization, which shortens the period over which the drying droplet is vulnerable [123]. Further, MD has been reported to entrap phenolic compounds through non-covalent secondary interactions—principally hydrogen bonding between the glycosidic and hydroxyl oxygens of the dextrin and the hydroxyl groups of the phenolics, together with hydrophobic interactions—rather than through selective covalent forces [124,125,126].

Similarly to our study, in a mulberry-leaf phenolic extract, different compound classes were retained to significantly different levels by spray drying, using MD as a wall material [127]; this was attributed to an increased susceptibility of phenolics to thermal degradation with an increase in the number of hydroxyl groups in the molecular structure. Moreover, Leyva-Porras et al. [128] reported that retention differences between spray-dried antioxidants using MD were dictated by the availability of functional groups for interaction with the carrier rather than by molecular size or steric effects. Regarding the examined compounds, oleocanthal and oleacein are reactive dialdehydes, existing in their hydrated and hemiacetal forms in the feed solution and are considered to be generally unstable and to degrade rapidly under thermal treatment [95]. In contrast, oleuropein aglycone is a hydroxytyrosol derivative, and the carbomethoxy group on the elenolic acid backbone makes it more chemically stable.

The produced encapsulated product was characterized by moderate moisture (equal to 11%) and hygroscopicity (0.23 g moisture/g dry product). These parameters indicated the product had the tendency to absorb moisture, indicating that special care should be taken during storage to maintain its stability, avoid microbial spoilage, and increase the shelf life of the powder. Moreover, its particle size distribution fell within the typical range of spray-dried (and/or encapsulated natural extracts) products of 10–100 µm [129].

Further, the flowability properties of the encapsulated product were determined, since they are strongly associated with the handling and processing of powders including mixing, pouring, packaging and storage [130,131,132]. The bulk density and tapped density of the sample, shown in Table 8, amounted to 0.32 g/mL and 0.65 g/mL, respectively. Also, the bulk and tapped density values of MD were found to be 0.46 ± 0.01 and 0.50 ± 0.02, respectively.

The terms of CI and HR, derived from the combination of bulk and tapped density, are used to predict powder flowability. Specifically, CI is associated with the resistance and stability of contact between particles, while HR is related to the friction between the particles [129]. According to Table 8 and Supplementary Table S6, the flowability of the spray-dried product is classified as “very poor” and also exhibits high cohesiveness. However, the angle of repose of the encapsulated product characterizes its flowability as “good”. Different flowability properties have been reported in the literature for natural extracts encapsulated by spray drying using MD as a wall material [133,134,135,136,137]. This variability could be attributed to the vast range of factors affecting the flowability of spray-dried phytochemical extracts, including particle size, powder composition, moisture content, surface and wall material properties, particle shape, roughness, hardness, and surface lubrication caused by moisture or fat [138].

It is important to mention that improved powder flowability is essential for the efficient production of nutraceutical and medicinal solid dosage formulations, facilitating uniform die filling, capsule filling, and tablet manufacturing while ensuring consistent weight, homogeneity, and bioactive content [121,139,140].

Regarding the color characteristics, the L* value of 45.26 indicated a relatively pale product, while the very low a* value (0.475) indicated a very light red hue. The b* value (1.85) reveals a very weak yellow component. Overall, the results show that the produced product exhibits moderate moisture and hygroscopicity, and significant retention of phenolic components, characteristics that make it technologically valuable and suitable for further exploitation as a functional ingredient.

**Figure 6:**
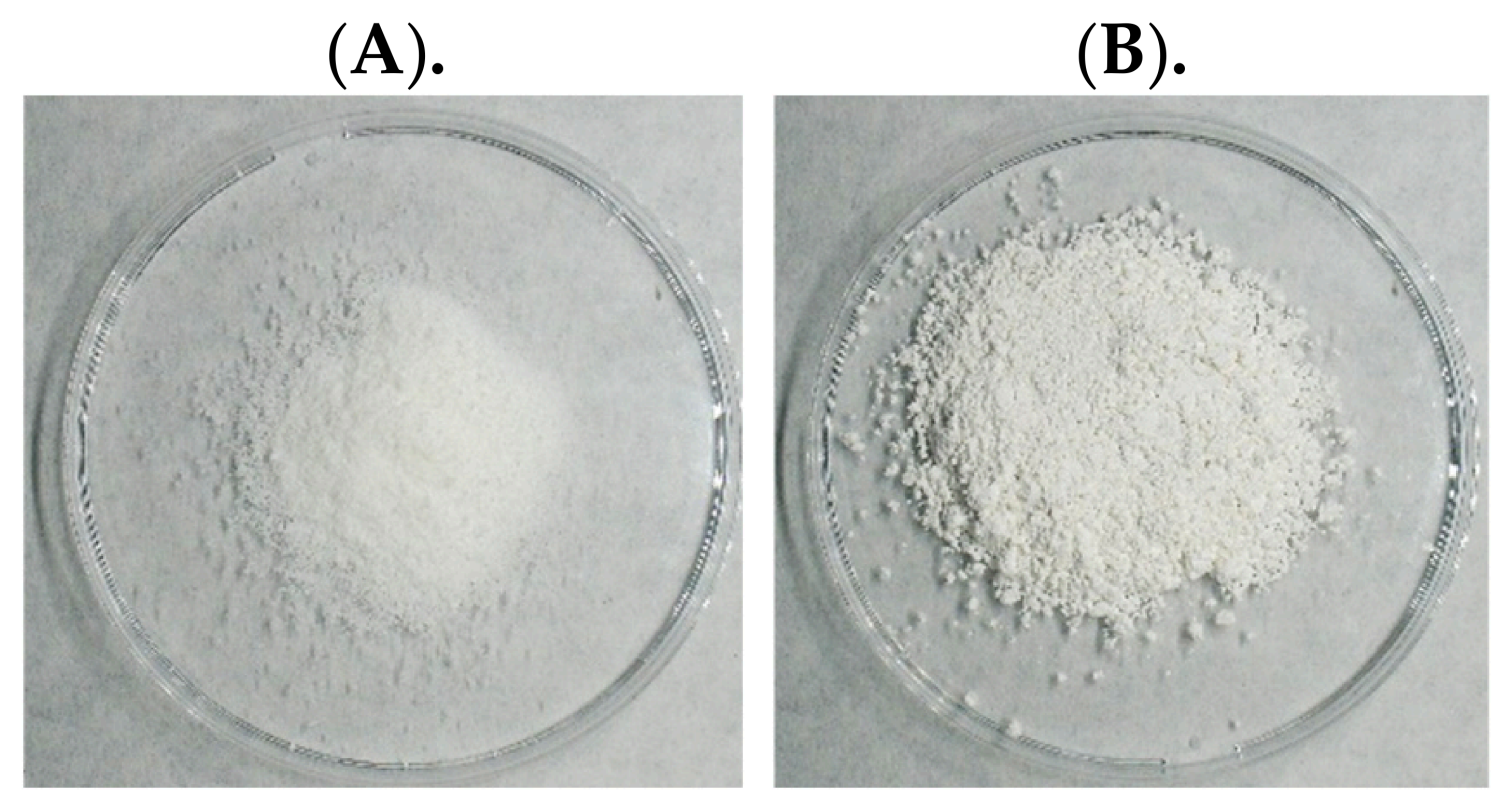
Morphological imaging of maltodextrin (**A**) and powder of the encapsulated EVOO phenolics (**B**) obtained by spray drying.

Figure 6 shows macroscopically that the produced powder exhibits a fine-grained and relatively homogeneous form, with a pale brownish color, which is probably related to both the natural shade of the phenolic extract and the presence of the encapsulating agent. By contrast, microscopically, aggregates of small particles are visible, a characteristic often found in spray-dried products, due to the rapid removal of water.

**Figure 7:**
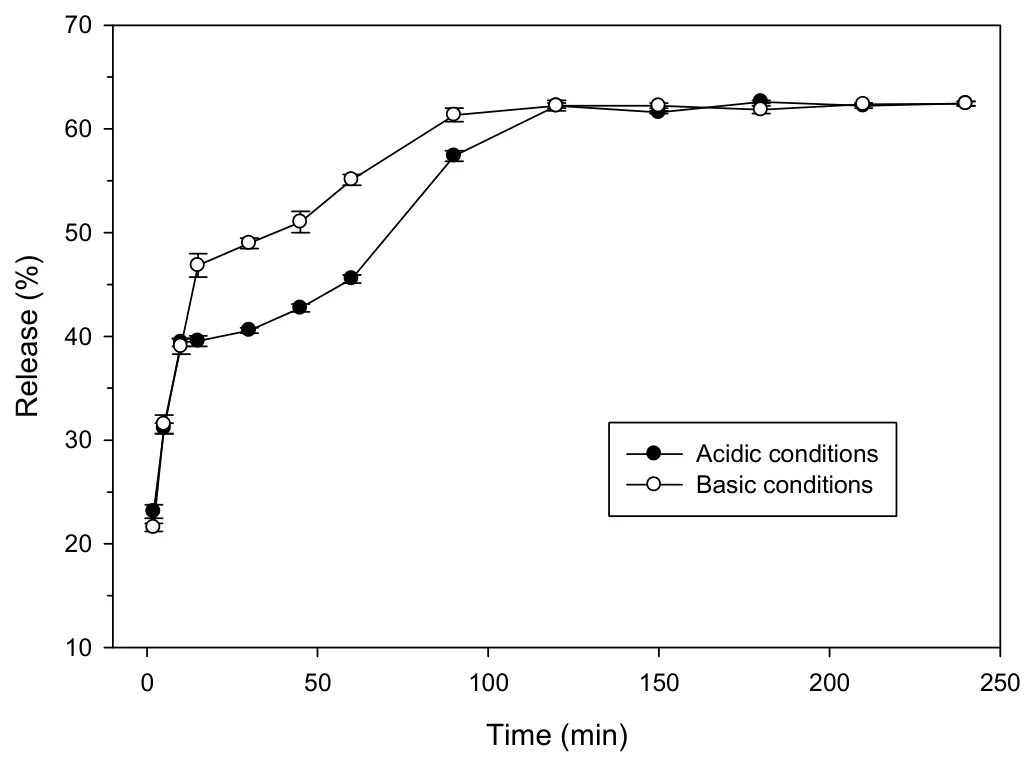
Percentage of release of TPC from polyphenol/maltodextrin powder. Release kinetics of phenolic compounds from the encapsulated extract in aqueous medium.

Controlled release is a critical parameter for encapsulated phenolic extracts, as it affects their bioavailability, stability and functional performance in potential food systems [141]. According to the results presented in Figure 7, three distinct stages of release are observed. In the first stage, the release of phenolic compounds increased significantly within the first 15 min, releasing 40 and 48% of TPC for acidic and basic conditions, respectively. During this stage, rapid hydration of the particle surface and direct diffusion of the most available phenolic compounds into the aqueous medium dominate. Thus, the increase can be attributed to the rapid dissolution or removal of compounds that are not fully encapsulated inside the encapsulating matrix or are located in sites directly accessible by the aqueous medium (surface or near the outer layer of the particles) [142].

In the second stage, a maximum release of 61.3–62.2% was observed at 120 and 90 min for acidic and basic conditions, respectively. In this stage, the release becomes slower; a significant percentage of the phenolic load is released in the aqueous medium, which was mainly controlled by the diffusion of the compounds through the encapsulating matrix, the swelling or relaxation of the matrix and the interactions between the core and the wall material [142]. Similar mechanisms have been described in encapsulated systems of phenolic compounds, where the release depends on the composition and solubility of the carrier, the particle size, the morphology of the matrix and the hygroscopic behavior of the product [142,143]. Basic conditions exhibited an increased first-stage release of phenolic compounds and a shorter second stage compared to the acidic conditions, probably due to more efficient hydration of the maltodextrin matrix [144].

#### 2.3.1. FTIR Characterization of Encapsulated Phenolic Compounds with Spray Drying

**Figure 8:**
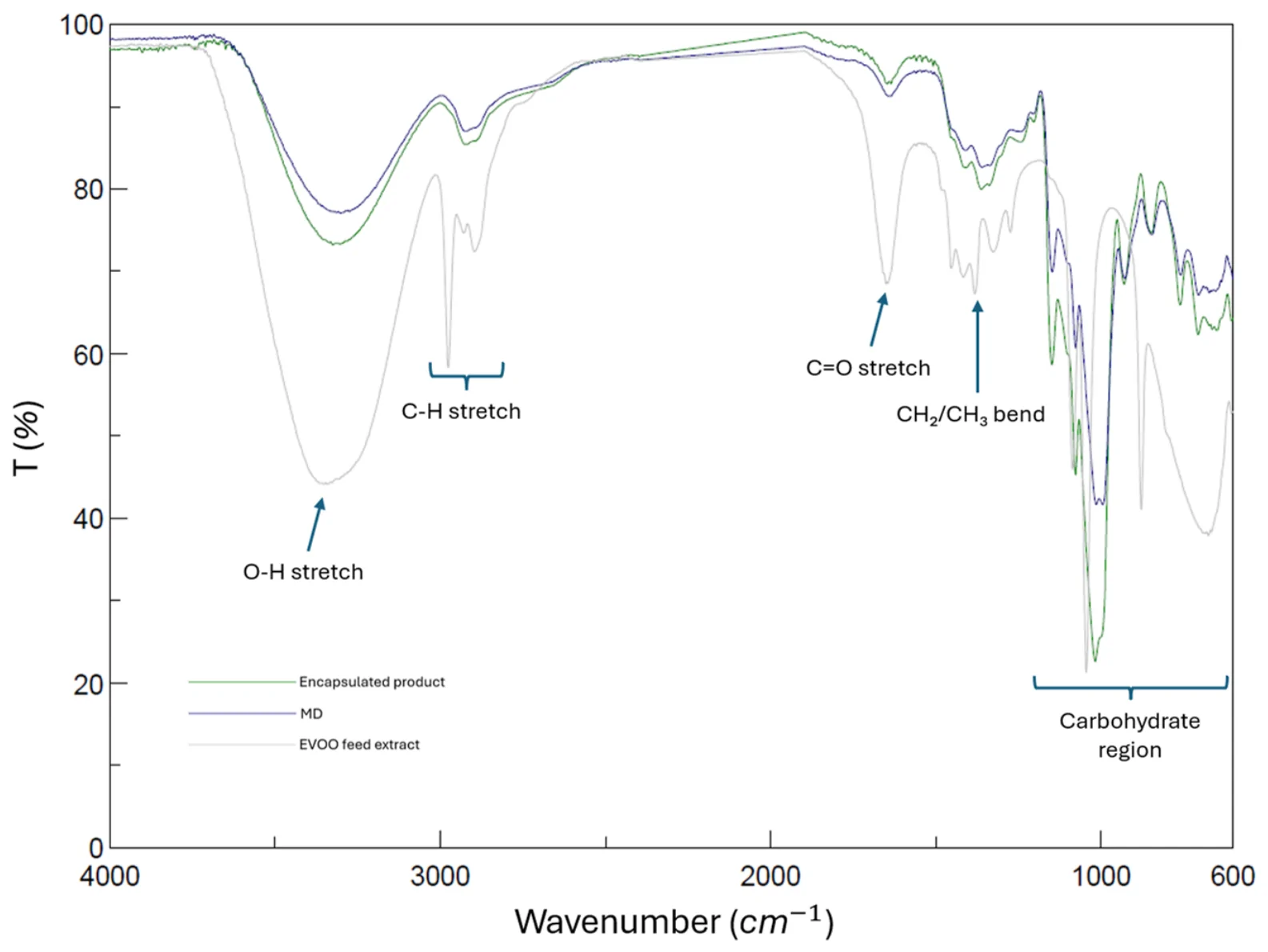
FT-IR spectra of the EVOO feed phenolic extract, the encapsulation carrier (maltodextrin) and the encapsulated sample produced via spray drying.

FT-IR spectroscopy analysis was employed in the present study with the aim of investigating the possible interactions between the feed olive oil extract and the encapsulation vehicle (maltodextrin). As shown in Figure 8, the spectrum of the encapsulated sample showed a high degree of overlap with the spectra of the encapsulation vehicle used. This observation is expected due to the high ratio of encapsulation medium to extract, which leads to the dominance of characteristic peaks of the carriers in the spectrum.

Firstly, a broad band observed at 3500–3000 cm^−1^ is attributed to the stretching vibrations of the free inter- and intra-molecular –OH groups, associated with the phenolic structure, as well as polysaccharides and absorbed water [145]. Peaks noted at 2921 and 2894 are associated with the (a-)symmetric stretching vibrations of the –CH group [146]. The peak detected at 1642 cm^−1^ is assigned to the stretching vibration of the carbonyl group (C=O), characteristic of maltodextrin, as well as the C–C bond and the bending of the hydroxyl group. Furthermore, a peak at 1406 cm^−1^ corresponds to bending vibrations of CH_2_ and CH_3_ groups, suggesting extensive carbon chain presence [147]. The absorption bands at 1145, 1077 and 998 cm^−1^ are associated with C–O and C–O–H bonds, whereas other minor bands in the 1450–1300 cm^−1^ region are attributed to –CH_2_, –CH, and =CH groups and assigned to the carbohydrate structure of maltodextrin and the presence of phenolic compounds [148,149,150,151].

Comparison of the FT-IR spectra of the encapsulated product and the pure maltodextrin revealed some minor differences. A slight increase in the intensity of the broad O-H stretching band in the 3500–3000 cm^−1^ region was observed in the encapsulated extract. This may be related to the contribution of hydroxyl groups present in the phenolic compounds from the olive oil extract and/or to hydrogen bond formation within the encapsulation matrix. Furthermore, changes in the intensity and position of several absorption bands in the 1650–1000 cm^−1^ region were observed. In particular, blueshifts in the bands observed at 1642 and 1077 cm^−1^ may also indicate increased interactions between the extract constituents and maltodextrin through non-covalent (hydrogen bonding and van der Waals) forces.

Importantly, no new intense absorption bands were detected in the spectrum of the encapsulated sample compared with the spectra of the maltodextrin or EVOO concentrated water extract. This finding suggests that the encapsulation process did not result in the formation of new covalent chemical bonds. Instead, the observed spectral changes, together with the overall similarity between the spectra, support the physical incorporation of the EVOO phenolic extract within the maltodextrin matrix.

#### 2.3.2. Folin–Ciocalteu and ^1^H-NMR Analysis of Encapsulated Phenolic Compounds

As seen in Table 5, the amount of the major secoiridoids determined was further slightly reduced in this stage; however, in a pattern similar to previous stages for all individual phenolics. The final recovery of EVOO phenols in the third step of encapsulation was 84.24%. A range from 50% to over 90% has been reported for recoveries of phenolic compounds from extracts of olive phenolics using spray drying and maltodextrin as a spray-drying carrier [152,153,154].

This indicated that, from a kilogram of EVOO subjected to water extraction and through the subsequent two steps (XAD-7HP recovery and spray-drying encapsulation), 25.31% would end up as the encapsulated material (Table 5). According to the ^1^H-NMR results for the phenolic profile of the encapsulated product, higher yields of oleocanthal and oleacein than of ligstroside and oleuropein aglycone were observed (Table 5). Nevertheless, all four major secoiridoids were retained in substantial amounts, indicating that the spray-drying process largely preserved the phenolic profile of the feed solution.

Finally, even though the final yield appears to be rather small, it is important to consider that water treatment of EVOO results in an olive oil that is still high in phenolics and can still be used as such. Further, considering that the European Food Safety Authority’s health claim refers to ≥5 mg of hydroxytyrosol and its derivatives, the quantity of encapsulated EVOO phenols would result in an appreciable number of 5 mg aliquots (EU health claim (Regulation (EU) No 432/2012) [11]. Moreover, the proposed extraction, recovery, and encapsulation process relies on green and sustainable principles, employing an aqueous NaCl solution as the extraction medium and regenerable XAD-7HP resin for phenolic recovery, thereby minimizing organic solvent consumption and enabling repeated resin reuse. The resulting phenolic-rich powder, characterized by high concentrations of olive oil phenolics, represents a promising constituent for the development of innovative nutraceutical supplements and/or medicinal formulations.

## 3. Materials and Methods

### 3.1. Chemicals

All reagents were of analytical grade. Ethanol and n-hexane were supplied by Chem-Lab (Zedelgem, Belgium). The HPLC mobile phase was prepared using HPLC-gradewater, methanol, and acetonitrile from Chem-Lab (Zedelgem, Belgium). Folin–Ciocalteu’s phenol reagent, sodium carbonate, glacial acetic acid, syringic acid, DPPH (2,2-diphenyl-picrylhydrazyl) and HPLC standards (hydroxytyrosol, tyrosol, vanillin, p-coumaric acid, ferulic acid, caffeic acid, ligstroside aglycone, oleuropein, oleacein, oleocanthal, cinnamic acid, verbascoside, luteolin, apigenin, syringic acid) were all purchased from Sigma-Aldrich (St. Louis, MO, USA). A Kern 770 balance (Balingen, Germany) was used to prepare all liquid solutions gravimetrically, with a precision of ± 0.0001 g. Syringaldehyde (98% purity), used as internal standard (IS), and chloroform-d for ^1^H-NMR measurements were purchased from Sigma-Aldrich (St. Louis, MO, USA). Amberlite resins (XAD 4, XAD 7HP, and XAD 16N) were purchased from Sigma-Aldrich (St. Louis, MO, USA); the physicochemical properties are summarized in Table 9.

### 3.2. Olive Oil Sample

The commercial high-phenolic EVOO sample used in this study was obtained from olives (*Olea europaea* L.) of the cv. *Koroneiki*, harvested and extracted during the 2025–2026 harvest season in Kalamata, Greece, EU. The EVOO satisfied the European Union regulatory standards for EVOOs and was marketed as a “polyphenol-rich” olive oil, possessing the established health claim under Regulation (EU) No 432/2012 [11]. Prior to further experimentation, the phenolic content of the EVOO was analyzed by employing the International Olive Council (IOC) chromatographic method [24], the colorimetric Folin–Ciocalteu method [155,156], and quantitative ^1^H-NMR spectroscopy [84].

### 3.3. Green Extraction of Phenolic Compounds

Extra virgin olive oil and aqueous NaCl solutions at the appropriate concentrations and ratios specified by the experimental design (Table 1) were mixed by vortexing for 3 min. The mixtures were then sonicated for 15 min at 25 °C, at a maximum power of 1200 W and ultrasonic frequency of 35 kHz with a Bandelin Sonorex Digiplus water bath (Berlin, Germany). Subsequently, they were centrifuged for 15 min at 9000 rpm (Hettich Universal, Tuttlingen, Germany), and the two phases formed were allowed to fully separate. Following centrifugation, the lower aqueous phase containing the extracted phenolic compounds was collected. The described extraction procedure was carried out in triplicate for each sample, and the pooled aqueous extracts were concentrated using adsorbent resins and analyzed in terms of their phenolic content and antioxidant activity.

#### 3.3.1. Experimental Design

To investigate the effects of the independent factors on the extraction process, a response surface methodology (RSM) approach was employed using Minitab Release 20 (Minitab, Inc., State College, PA, USA) software. A blocked face-centered central composite design (FC-CCD) was used to study the effects of salt content (%) in water (X_1_) and solvent/EVOO ratio (X_2_) on total phenolic content (Y_1_) and % radical scavenging activity (Y_2_) at three experimental levels coded as −1, 0, +1, where the axial distance (α) was set to 1. The selected factors and their ranges were determined based on preliminary experimentation. Six of the 14 experimental runs that made up FC-CCD were carried out at the center point and duplicated for error estimation (Table 1). To optimize the impact of unexplained variability in observed responses resulting from unrelated causes, experiments were randomized.

A second-order polynomial model was fitted to the data:

Y = β_0_ + β_1_X_1_ + β_2_X_2_ + β_11_X_1_^2^ + β_22_X_2_^2^ + β_12_X_1_X_2_ + ε (1)

where Y is the response, X_1_ and X_2_ are coded variables, and β are the regression coefficients.

ANOVA was used to assess the quality of the fitted models, including the R^2^ values, *p*-values, and lack-of-fit. Terms with *p*-values < 0.050 were considered statistically significant.

#### 3.3.2. Optimization of Extraction

Minitab software (Release 20, Minitab, Inc., State College, PA, USA) was used to optimize multiple responses and determine the optimal combination of experimental conditions. The predicted optimal conditions were subsequently verified experimentally to assess the accuracy of the developed model for total phenolic content using the Folin–Ciocalteu method. The optimized sample was also evaluated for DPPH radical scavenging activity and phenolic profile by quantitative ^1^H-NMR spectroscopy.

### 3.4. Recovery of Phenolic Compounds

The optimized extract was subjected to adsorption and desorption experiments using three Amberlite macroporous resins (XAD 4, XAD 7HP, and XAD 16N) [40,41,42] to evaluate their capacity for phenolic compound recovery. Firstly, resin pretreatment was performed according to Zagklis et al. [36]. Briefly, the resins were initially soaked in acetone for 8 h under magnetic stirring to remove residual monomers and subsequently dried at room temperature. The dried resins were then rinsed with ethanol (5 mL/g resin), followed by washing three times with triple-distilled water. Finally, all resins were dried in a convective oven at 60 °C for 24 h and weighed until a constant weight was achieved for moisture content determination.

Following the static adsorption and desorption experiments, the resin exhibiting the best adsorption and desorption performance was selected for the subsequent experiments. A larger volume of olive oil was extracted under the determined optimum extraction conditions using aqueous NaCl solution, and the resulting extract was passed through a column packed with the selected Amberlite resin. The adsorption process was performed by loading the extract onto the resin bed, allowing the phenolic compounds to be retained by the resin.

Subsequently, for the adsorption step, the retained phenolic compounds were desorbed from the resin using the optimized ethanol/water mixture. A sufficient number of bed volumes was passed through the column to ensure complete desorption, which was confirmed by measuring the total phenolic content of each collected bed volume using the Folin–Ciocalteu method. The final eluate was also analyzed in terms of its phenolic profile by ^1^H-NMR.

#### 3.4.1. Static Adsorption and Desorption Evaluation

Pretreated 2.0 g of dry resin was mixed with 30 mL of extract in a 100 mL flask. All the flasks with stoppers were shaken in a water bath at 25 °C at a shaking speed of 120 rpm for 24 h. When adsorption reached equilibrium, the resin used was filtered, and the filtrate was used to determine the content of phenolic compounds. Subsequently, the resin was washed with 20 mL of distilled water, and then desorption was carried out with 40 mL of 50% ethanol, 75% ethanol and 100% ethanol in a 50 mL flask. Then, the desorption processes were conducted in a thermostatic oscillator at room temperature (25 °C) at the same shaking speed for 12 h. The phenolic content in the liquid phase was measured by means of the Folin–Ciocalteu colorimetric method.

(2) $$\mathrm{Adsorption}\;\mathrm{content}:\;Q_{e}=\frac{\left(C_{0}-C_{e}\right)\times V_{i}}{W}$$

(3) $$\mathrm{Adsorption}\;\mathrm{rate}:\;A=\frac{\left(C_{0}-C_{e}\right)}{C_{0}}\times 100\%$$

where $Q_{e}$ stands for the equilibrated adsorption content (mg/g dry resin). $C_{0}$ and $C_{e}$ refer to the initial and equilibrium concentrations (mg/mL), respectively. A is the adsorption rate (%). $V_{i}$ is the volume of the initial sample solution (mL). W is the weight of the dry resin tested (g). The desorption capacity was evaluated as follows:

(4) $$\mathrm{Desorption}\;\mathrm{rate}:\;D=\;\frac{\;C_{d\;}\times \;V_{d}}{\left(C_{0}\;-\;C_{e}\right)\;\times \;V_{i}}\;\times 100\%$$

(5) $$\mathrm{Desorption}\;\mathrm{content}:\;Q_{d}=\frac{\;C_{d\;}\times V_{d}}{W}$$

(6) $$\mathrm{The}\;\mathrm{recovery}:\;R=\frac{\;C_{d\;}\times V_{d}}{\;C_{0}\times V_{0}}\times 100\%$$

where D refers to the desorption rate (%). $C_{d\;}$ represents the concentration of the sample after desorption (mg/mL). $V_{d}$ is the volume of the desorption solution (mL). $Q_{d}$ refers to the desorption content (mg/g dry resin). R stands for the recovery (%).

#### 3.4.2. Adsorption Kinetics Test

The resin that exhibited the best performance in the static adsorption and desorption experiments was selected for the adsorption kinetics study. Briefly, 2 g of pretreated resin and 30 mL of the optimum extract were added to a 100 mL Erlenmeyer flask. The flask was placed on an orbital shaker at 120 rpm and 25 °C for 48 h. Aliquots of 100 μL were collected at predetermined time intervals (2, 5, 10, 15, 30, 45, 60, 90, 120, 150, 180, 210, 240, 300, 360, 420 and 580 min) and analyzed for total phenolic content using the Folin–Ciocalteu method. The adsorption kinetics were evaluated by fitting the experimental data to two kinetic models: the pseudo-first-order and pseudo-second-order models.

(7) $$\mathrm{Pseudo}-\mathrm{first}-\mathrm{order}\;\mathrm{model}:\;\ln(q_{e}-q_{t})=-k_{1}\times t+\ln q_{e}\;$$

(8) $$\mathrm{Pseudo}-\mathrm{second}-\mathrm{order}\;\mathrm{model}:\;\frac{1}{q_{t}}=\frac{1}{k_{2}\times q_{e}^{2}}\times \frac{1}{t}+\frac{1}{q_{e}}$$

(9) $$\mathrm{The}\;\mathrm{particle}\;\mathrm{diffusion}\;\mathrm{kinetic}\;\mathrm{equation}:\;q_{t}=k_{d\;}\times \sqrt{t}+C$$

where $q_{e}$ is the adsorption contents at equilibrium (mg/g). qt stands for the concentration level of phenolics absorbed at time t (mg/g). $k_{1}$, $k_{2}$ represent the rate constants of two kinds of kinetic models. $k_{d}$ refers to the rate constant of the particle diffusion kinetics model.

### 3.5. Spray Drying of Concentrated Extracts

The collected eluate obtained after the resin desorption process was subsequently subjected to rotary evaporation under vacuum until complete dryness. For the reconstitution step, a 17% (*w*/*v*) aqueous maltodextrin solution was prepared according to Kiritsakis et al. [45] to obtain the feed material for the following spray-drying process.

A laboratory-scale spray dryer (Büchi Mini Spray Dryer B-290, Büchi Labortechnik AG, Flawil, Switzerland) equipped with a two-fluid nozzle atomizer was used for the spray-drying process. The feed solution was introduced into the drying chamber using a peristaltic pump, while the drying air was heated and flowed concurrently with the atomized feed. The resulting powder was separated from the drying air using a cyclone separator and collected in the receiving vessel. The inlet drying air temperature was set at 150 °C, the aspirator rate was maintained at 100%, and the pump rate was set at 5%.

The encapsulation yield (Y) was calculated as an indicator of process performance. Yield was determined as the ratio of the dry solid mass of the recovered encapsulated product to the total solid mass initially subjected to spray drying:

(10) $$Y\;\left(\%\right)=\;\frac{\;dry\;solid\;mass\;of\;recovered\;encapsulated\;product}{total\;dry\;solid\;mass\;in\;the\;feed}\;\;\times 100$$

### 3.6. Characterization of the Encapsulated Products

Following production, all samples were collected and evaluated for their physicochemical properties and encapsulation performance. The analyses were performed immediately after sample preparation to minimize any potential changes in the characteristics of the products prior to characterization.

#### 3.6.1. Particle Size Distribution

The particle size distribution of the encapsulated powder and the maltodextrin used as the wall material in spray-drying process was analyzed using a SALD-7500 nano laser diffraction analyzer (SHIMADZU, Kyoto, Japan) operating in the range of 7 nm to 800 μm. The refractive index value of the samples was set at 1.35. The D50 value was also calculated to represent the particle diameters at which 50% of the total particle volume was smaller than the stated size.

#### 3.6.2. Flowability Properties

The bulk and tapped density of the spray-dried extract and the wall material (maltodextrin) were measured according to the method of Jinapong et al. [157], with minor modifications. Regarding bulk density, 10 g (M_1_) of sample was added into a tared graduated cylinder (50 mL), and the volume occupied by the powder (V_b_) was marked. Bulk density (ρ_bulk_) was calculated by the equation below:

(11) $$\rho_{bulk}=\;\frac{M_{1}}{V_{b}}$$

where $\rho_{bulk}$, $V_{b}$ and $M_{1}$ are the bulk density (g mL^−1^), mass of powder (g) and volume occupied (mL).

For tapped density measurement, the same amount of powder used for bulk density determination (10 g, M_2_) was kept in the tared 50 mL graduated cylinder, and the initial powder volume was recorded as (V_b_). The cylinder was then subjected to mechanical tapping using a volumeter (Model JEL ST 2; J. Engelsmann, Ludwigshafen, Germany) until no further change in volume was observed. The final volume occupied by the powder was recorded as (V_t_), and tapped density was calculated according to the following equation:

(12) $$\rho_{tapped}=\;\frac{M_{2}}{V_{t}}$$

where $\rho_{tapped}$, $V_{t}$ and $M_{2}$ are the tapped density (g mL^−1^), mass of powder (g) and volume occupied (mL).

For further investigation of the flowability and cohesiveness properties of the examined powders, the compressibility index (CI) [158], Hausner ratio (HR) [159] and angle of repose (θ) were determined; classification of expected flow character of the powders based on the CI, HR and angle of repose values are shown in Supplementary Table S6. The CI and HR values were calculated from the bulk (ρ_bulk_) and tapped (ρ_tapped_) densities of the powder as shown in Equations (13) and (14):

(13) $$CI=\frac{\left(\rho_{tapped}-\rho_{bulk}\right)}{\rho_{tapped}}\times 100\;$$

(14) $$HR=\frac{\rho_{tapped}}{\rho_{bulk}}$$

The angle of repose (θ) was measured according to the method of Zhao et al. [160] with minor modifications. A funnel was fixed vertically three centimeters above the plane on the testbed. The produced powder was continuously poured into the funnel until the formed cone contacted the funnel end. The angle of repose (θ) was calculated by Equation (15):

(15) $$\theta =2\times arctan(\frac{2R}{H})$$

where H (cm) and R (cm) were the height and radius of the powder cone, respectively.

#### 3.6.3. Color

The colors of the produced powder and maltodextrin were determined using a Minolta colorimeter (CR-410, Minolta, Japan). A white color standard was used for calibration and as a background for color measurements of the samples. The Hunter Lab color scale was used for measuring color values: $L^{*}$, the lightness variable; $a^{*}$, from green to red; and $b^{*}$, from blue to yellow. The total color difference (ΔE) of the spray-dried extract compared to maltodextrin was calculated as follows:

(16) $$\Delta E=\sqrt{{\left(L^{*}-L_{o}^{*}\right)}^{2}+{\left(a^{*}-a_{o}^{*}\right)}^{2}+{\left(b^{*}+b_{o}^{*}\right)}^{2}}$$

where $L_{o}^{*}$ was the lightness, $a_{o}^{*}$ was the greenness and $b_{o}^{*}$ was the yellowness of the maltodextrin.

#### 3.6.4. Moisture Content and Hygroscopicity

The moisture content of the encapsulated samples was determined on a wet basis by drying them in an oven at 105 °C until a constant weight was achieved. Following drying, the samples were cooled to room temperature in a desiccator and subsequently weighed.

For the determination of hygroscopicity, approximately 1 g of powder was evenly distributed in a porcelain capsule to maximize the surface area exposed to the surrounding air. The samples were then placed in a desiccator maintained at 23 °C and 76% relative humidity, with the desired humidity level established using a nitric acid (HNO_3_) solution. Moisture sorption kinetics were evaluated by recording changes in sample weight at 10 min intervals. Hygroscopicity was expressed as the increase in mass per gram of powder solids, reflecting the moisture uptake capacity of the powders under controlled relative humidity conditions.

#### 3.6.5. FTIR Spectroscopy

Attenuated total reflection (ATR) FT-IR spectra for the encapsulated product, maltodextrin and concentrated phenolic extract were acquired using a 6700 IR (Jasco, Essex, UK) spectrometer equipped with a DLaTGS detector and a high-throughput Single-Reflection ATR with a diamond crystal and accompanied by Spectra Manager software (version 2) (Jasco, Essex, UK). FTIR spectra of each sample were recorded in transmittance mode over the wavenumber range of 4000–600 cm^−1^, using a spectral resolution of 4 cm^−1^ and 64 scans per spectrum. Ten spectra were acquired for each sample to ensure measurement reproducibility. The original spectra were corrected with the aid of Spectra Manager software (V.2.15.01, JASCO, Great Dunmow, UK).

#### 3.6.6. Release of Phenolic Compounds Under Acidic and Basic pH Conditions

The release behavior of phenolic compounds from the encapsulated phenolic extract was evaluated under acidic and basic pH conditions. Two aqueous media were prepared and adjusted to pH 1.2 and pH 7.4; encapsulated phenolic extract (2 g) was dispersed in the corresponding release medium and maintained at 37 °C under continuous shaking (100 rpm). Aliquots of 100 μL were withdrawn at predetermined time intervals (2, 5, 10, 15, 30, 45, 60, 90, 120, 150, 180, 210, and 240 min) to monitor the release of phenolic compounds over time. The obtained samples were analyzed for their phenolic content, and the percentage of phenolic compounds released was calculated relative to the total phenolic content initially present in the encapsulated phenolic extract. The release profiles obtained under the two pH conditions were compared to evaluate their effect on the release behavior of the encapsulated phenolic compounds. The experiment was run in triplicate.

### 3.7. Analyses

#### 3.7.1. Determination of Total Phenolic Content

The Folin–Ciocalteu reagent, as reported by Singleton et al. [155] and Scalbert et al. [156], was used with some modifications to colorimetrically determine the total phenolic content. Water was used as a control, and 100 μL was diluted with 2.9 mL of deionized water and mixed with 0.25 mL of Folin–Ciocalteu reagent and, after 1 min, with 0.75 mL of Na_2_CO_3_ (20% *w*/*v*), then stored in a dark place at room temperature (at 20 °C) for 60 min. A Genesys 180 UV-VIS spectrophotometer (Thermo Fisher Scientific, Waltham, MA, USA) was used to measure the solution’s absorbance at 760 nm. The linear regression equation of standard curve (y = 0.0301x + 0.0261, R^2^ = 0.990) was used to calculate total phenolic compounds (TPCs), which were then expressed as tyrosol equivalents (Tyr Eq. mg/kg). When required, the samples were diluted using an appropriate ethanol/water mixture; all EVOO extract analyses were conducted in triplicate (*n* = 9); and the reagents were handled in the coldest, darkest feasible conditions.

#### 3.7.2. Determination of Radical Scavenging Activity

With minor adjustments, the antiradical properties of the samples were determined using DPPH as a free radical according to Brand-Williams et al. [161] and Nenadis and Tsimidou [162]. In a test tube, 3.9 mL of DPPH methanolic solution (100 μM) was mixed, and 0.1 mL of water was used as a control. After that, the tubes were vortexed and left at room temperature (at 20 °C) for 30 min in a dark environment. A Genesys 180 UV-VIS spectrophotometer (Thermo Fisher Scientific, Waltham, MA, USA) was used to measure the absorbance of the solution at 517 nm. The radical scavenging antioxidant activity of the olive oil extracts, expressed as (%) values (%*RSA*), was determined by using the following formula (after correction with appropriate blanks):

(17) $$\%RSA=\frac{\left({ABS}_{517\left(t=0\right)}-{ABS}_{517\left(t\right)}\right)}{{ABS}_{517\left(t=0\right)}}\times 100$$

where *ABS* refers to the absorbance of a blank sample (*t* = 0) and to the absorbance of an analyzed sample (*t*). All measurements were carried out in triplicate (*n* = 9).

#### 3.7.3. HPLC-DAD Phenolic Profile Analysis

The MeOH/water extract of the EVOO was analyzed using an HPLC-DAD system (ECOM, Prague, Czech Republic) with a Supelco Discovery HS C18 column (5 μ, 150 × 4.6 mm I.D., St. Louis, MO, USA) at 25 °C, according to the official method of the International Olive Oil Council for the quantification of phenolics in olive oil [24]. The elution was carried out in gradient mode using a three-phase organic solvent mixture composed of water acidified with 0.2% acetic acid (solvent A), methanol (solvent B), and acetonitrile. A linear gradient ran from 96% (A), 2% (B), and 2% (C) to 50% (A), 25% (B), and 25% (C) over 40 min; it changed to 40% (A), 30% (B), and 30% (C) for 5 min; and then it changed to 0% (A), 50% (B), and 50% (C) for 25 min, followed by a 12 min re-equilibration to the initial solvent composition. The mobile phase flow rate was 1 mL/min, and each sample had an injection volume of 20 μL. All phenolic compounds were identified by comparing retention times to those of standards (hydroxytyrosol, tyrosol, vanillin, p-coumaric acid, ferulic acid, ligstroside aglycone, oleuropein, oleacein, oleocanthal, cinnamic acid, apigenin, luteolin, verbascoside, pinoresinol and syringic acid as internal standard). The HPLC analyses of EVOO extract were carried out in triplicate (*n* = 3).

#### 3.7.4. NMR Analysis

The method was adapted from Karkoula et al. [84]; olive oil (5.0 g) was mixed with cyclohexane (20 mL) and acetonitrile (25 mL). The mixture was homogenized using a vortex mixer for 30 s and centrifuged at 4000 rpm for 5 min. A part of the acetonitrile phase (25 mL) was collected, mixed with 1.0 mL of a syringaldehyde solution (0.5 mg/mL) in acetonitrile, and evaporated under reduced pressure using a rotary evaporator (Buchi, Flawil, Switzerland).

The residue of the above procedure was dissolved in CDCl_3_ (750 μL), and an accurately measured volume of the solution (550 μL) was transferred to a 5 mm NMR tube. The ^1^H-NMR (500.1 MHz) spectra were recorded on an AGILENT DD2 500 spectrometer (Agilent Technologies, Santa Clara, CA, USA). Chemical shifts are reported in δ (ppm) values relative to TMS (7.26 ppm for CDCl_3_). Typically, 50 scans were collected into 32K data points over a spectral width of 0–16 ppm with a relaxation delay of 1 s and an acquisition time of 1.7 s. Prior to Fourier transformation (FT), an exponential weighting factor corresponding to a line broadening of 0.3 Hz was applied. The spectra were phase corrected and integrated automatically using MestReNova (version 12.0.0-20080).

### 3.8. Statistical Analysis

Statistical analysis was performed by analysis of variance (ANOVA) with Minitab Release 20 (Minitab, Inc., State College, PA, USA) software. All tests were performed in triplicate, and the results were expressed as means ± standard deviation (SD).

## 4. Conclusions

Water extraction techniques were successfully used and optimized to extract phenolic compounds from a polyphenol-rich EVOO matrix. A maximum water/EVOO ratio of 9.6:1 (*v*:*v*) was determined to be optimal for maximizing extraction yield. Salt at 3% was used to avoid the formation of an emulsion while still providing similar phenolic yields to the optimized extract.

Using water in the extraction process is considered not only green but also industry-friendly. It should be noted that using water to extract phenolics from high-phenolic EVOO can achieve a yield of up to 39%. By using food-grade water, NaCl, and only physical processes (sonication, centrifugation) that do not include any non-food ingredients, the EVOO remains edible, preserving its quality parameters and most of its phenolics while exhibiting less but significant organoleptic traits. Compositionally, the extract was rich in oleocanthal–oleacein and moderate in oleuropein–ligstroside aglycones. For phenolic recovery, XAD-7HP resin and 75% aqueous ethanol eluent were implemented with low total phenolic losses, while the composition remained virtually unchanged. The phenolics were also encapsulated with low phenolic losses. The produced powder showed a typical phenolic release pattern and had moderate hygroscopicity, indicating the need for care in its storage. The overall yield of phenolic compounds per kg of EVOO was 25%, which is considered moderate. It should be noted that the resin may be regenerated for recurrent use, making the entire process environmentally friendly and scalable. Future research on shelf-life and storage stability would supplement the current findings, offer insight into the encapsulated product’s long-term preservation, and evaluate its practical usefulness.

A sustainable and environmentally friendly quantitative procedure for the extraction and encapsulation of olive oil polyphenols could potentially be used in the development of unique functional foods and/or dietary supplements aimed at key diseases that polyphenols have been shown to prevent or treat.

## Figures and Tables

**Table 1:** Experimental design of the two-factor face-centered central composite design (FC-CCD).

**Factor**	**Variable**	**Levels**				
		**Coded Values ^1^**				
		−a	−1	0	1	+a
		**Actual Values**				
Salt content (%)	X_1_	0	0	15	30	30
Water/EVOO ratio	X_2_	1	1	5.5	10	10
		Run Order	X_1_	X_2_	Coded values	
		1	15	5.5	0	0
		2	15	5.5	0	0
		3	15	10	0	1
		4	30	5.5	1	0
		5	15	5.5	0	0
		6	0	5.5	−1	0
		7	15	1	0	−1
		8	15	5.5	0	0
		9	15	5.5	0	0
		10	15	5.5	0	0
		11	0	10	−1	1
		12	30	1	1	−1
		13	0	1	−1	−1
		14	30	10	1	1

^1^ Coded value = [actual level − (high level + low level)/2]/(high level − low level)/2.

**Table 2:** Quadratic regression models and statistical parameters for the responses (total phenolic compounds—TPC, antiradical scavenging activity—%RSA).

Sample	2nd Order Polynomial Equation	Regression (*p*-Value)	R^2^	R^2^ (Adjusted)	R^2^ (Predicted)	Lack of Fit
TPC	y = 4.6 − 5.2296X_1_ + 49.71X_2_ + 0.1637X_1_^2^ − 2.570X_2_^2^ − 0.7274X_1_X_2_	<0.001	99.03	98.21	93.95	0.051
%RSA	y = 105.39 − 3.028X_1_ + 15.97X_2_ + 0.1019X_1_^2^ + 1.200X_2_^2^ − 0.2272X_1_X_2_	<0.001	98.43	97.08	91.48	0.154

**Table 3:** Experimental and predicted values for total phenolic content (TPC, mg Tyr. Eq./kg EVOO) and radical scavenging activity (%RSA).

Run	Salt Content (%)-X_1_	Ratio (Water/EVOO)-X_2_	Experimental Values		Predicted Values	
			TPC (mg Tyr Eq./kg EVOO)	%RSA	TPC (mg Tyr Eq./kg EVOO)	%RSA
1	15.0	5.5	92.90 ± 0.46	13.66 ± 1.40	98.64	12.60
2	15.0	5.5	104.68 ± 0.73	12.40 ± 0.26	98.64	12.60
3	15.0	10.0	109.60 ± 1.33	3.80 ± 0.60	93.94	9.07
4	30.0	5.5	78.64 ± 1.00	7.46 ± 0.13	70.72	17.23
5	15.0	5.5	101.12 ± 0.46	13.40 ± 0.33	98.64	12.60
6	0.0	5.5	219.96 ± 1.46	49.53 ± 1.40	200.24	53.83
7	15.0	1.0	11.23 ± 0.13	55.93 ± 0.60	0.00	64.72
8	15.0	5.5	94.36 ± 0.64	19.13 ± 0.60	98.64	12.60
9	15.0	5.5	97.28 ± 0.64	7.86 ± 0.53	98.64	12.60
10	15.0	5.5	90.43 ± 0.18	15.66 ± 0.33	98.64	12.60
11	0.0	10.0	229.70 ± 0.17	68.80 ± 2.26	244.63	65.63
12	30.0	1.0	13.23 ± 0.11	92.33 ± 0.60	20.42	84.68
13	0.0	1.0	38.65 ± 0.03	95.53 ± 0.33	51.74	90.61
14	30.0	10.0	7.89 ± 1.11	4.26 ± 0.13	16.92	0.00

**Table 4:** Experimental validation data for the predicted values under optimal extraction conditions.

Sample	Salt Content (%)-X_1_	Ratio (Water/EVOO)-X_2_	Response	Predicted Value	Experimental Value	%CV	%RSA
EVOO	0	9.6	TPC	244.91	252.12 ± 0.384	0.15	70.06 ± 0.60

**Table 5:** Individual phenolic concentrations, total phenolics based on NMR analysis or Folin–Ciocalteu method for EVOO, optimum water extract, XAD-7HP-recovered extract and spray-dried encapsulated phenolics.

Phenolics (mg/kg)	Initial EVOO	Water Extract ^x^	Extract After Desorption	Spray Dried Powder
Oleocanthal	297.43 ± 3.34	160.41 ± 3.35	149.51 ± 4.16	994.50 ± 12.78
Oleacein	274.40 ± 7.03	137.20 ± 3.51	127.63 ± 8.93	701.67 ± 7.41
Oleuropein aglycone	228.47 ± 4.15	33.23 ± 5.18	33.88 ± 6.54	390.47 ± 8.31
Ligstroside aglycone	250.61 ± 3.97	79.56 ± 4.18	76.13 ± 3.49	598.06 ± 10.18
Total Phenolics (NMR)	1050.92 ± 3.87	410.41 ± 3.07	387.15 ± 4.93	2684.71 ± 8.64
TPC (Folin–Ciocalteu)	728.51 ± 1.17	276.50 ± 0.83	260.29 ± 1.39	2179.65 ± 6.38

^x^ Water extract obtained at water-to-EVOO ratio of 9.6 and salt content of 3%.

**Table 6:** Static adsorption and desorption of extracted olive oil polyphenols on selected XAD resins (Mean ± SD, *n* = 3).

Resin	Q_e_ (mg/g)	A (%)	Q_d_ (mg/g)	D (%)	R (%)
XAD-4	3.21 ± 0.04	77.55 ± 1.10	2.11 ± 1.54	65.77 ± 1.54	50.99 ± 0.46
XAD-7HP	4.03 ± 0.01	97.39 ± 0.25	2.69 ± 0.39	66.59 ± 0.17	65.20 ± 0.33
XAD-16N	3.90 ± 0.01	94.08 ± 0.35	2.35 ± 0.12	60.46 ± 0.12	56.88 ± 0.33

Q_e_ stands for the equilibrated adsorption content (mg/g dry resin). A is the adsorption rate (%). Q_d_ refers to the desorption content (mg/g dry resin). D refers to the desorption rate (%); R stands for the recovery (%).

**Table 7:** Kinetic model fitting equations and model dynamic parameters for XAD-7HP.

Dynamic Model Kinetic Equations	Parameters	R^2^
The pseudo-first-order model: $ln(q_{e}\;\;-\;q_{t})\;=\;-k_{1}\times t\;+\;ln(q_{e})$	$q_{e}$ = 2.313 k_1_ = 0.0392	0.9754
The pseudo-second-order model $1/q_{t}\;=\;\;1/(k_{2}\;\;\times \;q_{e}^{2})\;\;\times \;\;1/t\;\;+\;\;1/q_{e}$	$q_{e}$ = 4.18 k_2_ = 0.0492	0.9997
Intra-particle diffusion model $q_{t}=\;k_{d}\times \;\surd t\;\;+C_{i}$	$k_{d}$ = 0.1362 $C_{i}$ = 2.8443	0.9644

$q_{e}$: the adsorption contents at equilibrium (mg/g). qt stands for the concentration of phenolic absorbed at time t (mg/g). $k_{1}$, $k_{2}$: the rate constant of two kinds of kinetic models, respectively. $k_{d}:$ the rate constant of the particle diffusion kinetics model.

**Table 8:** Physicochemical characteristics of the maltodextrin carrier and the encapsulated phenolic extract obtained by spray drying.

Physical Properties	Maltodextrin			Encapsulated Phenolics		
Moisture content (%)	-			11.00	±	0.20
Hygroscopicity (g H_2_O/g solids)	-			0.23	±	0.05
ρ_b_ (bulk density), (g/mL)	0.46	±	0.01	0.32	±	0.01
ρ_t_ (tapped density), (g/mL)	0.50	±	0.01	0.65	±	0.02
HR (Hausner ratio)	1.09	±	0.01	2.03	±	0.03
CI (Carr index), (%)	8.03	±	1.06	50.81	±	0.81
Angle of repose (°)	38.95	±	0.85	35.64	±	0.39
Color, Lightness (L*)	44.45	±	0.67	45.26	±	0.01
(a*)	-0.03	±	0.02	0.475	±	0.01
(b*)	2.897	±	0.09	1.85	±	0.01
DE	-			1.17	±	0.01
Particle size (µm)	17.96	±	0.43	6.34	±	0.48

**Table 9:** Physicochemical properties of the resins used in this study.

Resin	Chemical Matrix	Polarity	Dry Density (vs. Wet) (g/mL)	Surface Area (Sq. m/g)	Pore Diameter, Average (Angstroms)	West Mesh Size (Nominal)	Pore Volume (mL/g)
XAD-4	Styrene-divinylbenzene	Nonpolar	1.08 (1.02)	725	50	20 to 60	0.98
XAD-7HP	Acrylic ester	Moderately polar	1.24 (1.05)	450	90	20 to 60	1.14
XAD-16N	Styrene-divinylbenzene	Nonpolar	1.08 (1.02)	900	100	20 to 60	1.82

## Data Availability

The data presented in this study are available on request from the corresponding author. The data are not publicly available due to privacy restrictions.

## Supplementary Materials

The following supporting information can be downloaded at https://www.mdpi.com/article/10.3390/molecules31183298/s1: Figure S1: Residual diagnostic plots for the total phenolic content (TPC, mg Tyr. Eq./kg EVOO) second-order polynomial model fitted to the face-centered central composite design (FC-CCD). (A) Normal probability plot of residuals; (B) residuals versus fitted values; (C) histogram of residuals; and (D) residuals versus observation order (*n* = 14 runs); Figure S2: Residual diagnostic plots for the radical scavenging activity (%RSA, mg Tyr. Eq./kg EVOO) second-order polynomial model fitted to the face-centered central composite design (FC-CCD). (A) Normal probability plot of residuals; (B) residuals versus fitted values; (C) histogram of residuals; and (D) residuals versus observation order (*n* = 14 runs); Figure S3: Correlation graph between total phenolic content (TPC, mg Tyr. Eq./kg EVOO) and radical scavenging activity (%RSA) of the aqueous EVOO extracts across the 14 runs of the face-centered central composite design (FC-CCD) (see Table 3). Pearson’s correlation coefficient (r = −0.041; 95% CI: −0.559, 0.501) indicates no significant linear correlation between the two responses; Figure S4: HPLC-DAD chromatogram of the polyphenols extracted with 80% Methanol/H2O (*v*/*v*) of EVOO, namely: Hydroxytyrosol, tyrosol, syringic acid (IS), vanillin, p-coumaric acid, caffeic acid, ferulic acid, verbascoside, oleacein, oleuropein, pinoresinol, ligstroside aglycone, oleocanthal, luteolin, cinnamic acid, and apigenin; Figure S5: Aldehydic region of the 1H-NMR spectrum of EVOO, showing the signals used for the quantification and integration of the internal standard syringaldehyde (9.82 ppm), ligstroside aglycone (9.52 ppm), oleuropein aglycone (9.50 ppm), oleocanthal (9.23 ppm), and oleacein (9.19 ppm); Table S1: Analysis of variance (ANOVA) for the second-order polynomial models fitted to the total phenolic content (TPC, mg Tyr. Eq./kg EVOO) and radical scavenging activity (%RSA) responses of the blocked face-centered central composite design (FC-CCD); Table S2: Quality parameters of initial and residual polyphenolic EVOO following water extraction under optimized conditions; Table S3: Single-factor experimental data for the effect of salt content on the total phenolic content (TPC, mg Tyr. Eq./kg EVOO) and radical scavenging activity (%RSA) of aqueous EVOO extracts at a fixed water/EVOO ratio of 5. Salt content varied from 0 to 30% (*w*/*w*). Experimental values are expressed as mean ± standard deviation of triplicate measurements. Predicted values were obtained from the second-order polynomial models fitted to the FC-CCD (Table 1). The relative error (%) between experimental and predicted values was calculated as (experimental − predicted)/predicted × 100; Table S4: Parameters of linear regression, LOD and LOQ for phenolic compounds by HPLC analysis; Table S5: HPLC-DAD profile analysis of the phenolic compounds polyphenolic EVOO used for water extraction; Table S6: Flowability classification of powders according to their compressibility index, Hausner ratio and angle of repose values.
