# Supplementary material for: Enhancing the Phenolic Value of Polyphenol-Rich Extra Virgin Olive Oils Through Optimized Water Extraction, Macroporous Resin Recovery and Spray Drying
Source: Molecules. 2026 Sep 17;31(18):3298. doi: 10.3390/molecules31183298 (PMC13609994; doi:10.3390/molecules31183298)
Supplement: Supplementary file 1 [file molecules-31-03298-s001.zip › molecules-4505995-supplementary.pdf]

Electronic Supplementary Materials

Enhancing the Phenolic Value of Polyphenol-Rich Extra Virgin Olive Oils Through Optimized Water Extraction, Macroporous Resin Recovery and Spray Drying

Athanasios Gerasopoulos [1], Kyriakos Kachrimanis [2,3] and Diamanto Lazari [1,*]

[1] Laboratory of Pharmacognosy, School of Pharmacy, Aristotle University of Thessaloniki, 54124 Thessaloniki, Greece

[2] Department of Pharmaceutical Technology, School of Pharmacy, Aristotle University of Thessaloniki, 54124 Thessaloniki, Greece

[3] Center for Interdisciplinary Research & Innovation, Aristotle University of Thessaloniki, 54124 Thessaloniki, Greece

Table S1. Analysis of variance (ANOVA) for the second-order polynomial models fitted to the total phenolic content (TPC, mg Tyr. Eq./kg EVOO) and radical scavenging activity (%RSA) responses of the blocked face-centered central composite design (FC-CCD).

		TPC				%RSA			
Source	DF	Adj SS	Adj MS	F-Value	P-Value	Adj SS	Adj MS	F-Value	P-Value
Model	6	58186.7	9697.8	119.57	0.000	13925.8	2320.97	73.06	0.000
Blocks	1	850.6	850.6	10.49	0.014	176.8	176.80	5.57	0.050
Linear	2	38611.9	19306.0	238.04	0.000	6653.6	3326.81	104.72	0.000
Salt (% w/w)	1	25161.5	25161.5	310.24	0.000	2009.3	2009.34	63.25	0.000
Water:EVOO ratio	1	13450.5	13450.5	165.84	0.000	4644.3	4644.27	146.19	0.000
Square	2	8397.1	4198.5	51.77	0.000	4779.8	2389.89	75.23	0.000
Salt (% w/w)*Salt (% w/w)	1	3701.7	3701.7	45.64	0.000	1433.6	1433.63	45.13	0.000
Water:EVOO ratio*Water:EVOO ratio	1	7388.4	7388.4	91.10	0.000	1609.4	1609.41	50.66	0.000
2-Way Interaction	1	9642.7	9642.7	118.90	0.000	940.4	940.44	29.60	0.001
Salt (% w/w)*Water:EVOO ratio	1	9642.7	9642.7	118.90	0.000	940.4	940.44	29.60	0.001
Error	7	567.7	81.1			222.4	31.77		
Lack-of-Fit	3	471.0	157.0	6.49	0.051	154.9	51.63	3.06	0.154
Pure Error	4	96.7	24.2			67.5	16.87		

	TPC		%RSA
Total	13	58754.4	14148.2

DF, degrees of freedom; Adj SS, adjusted sum of squares; Adj MS, adjusted mean square; F-value, Fisher's ratio; P-value, probability value. Terms with p < 0.050 were considered statistically significant.

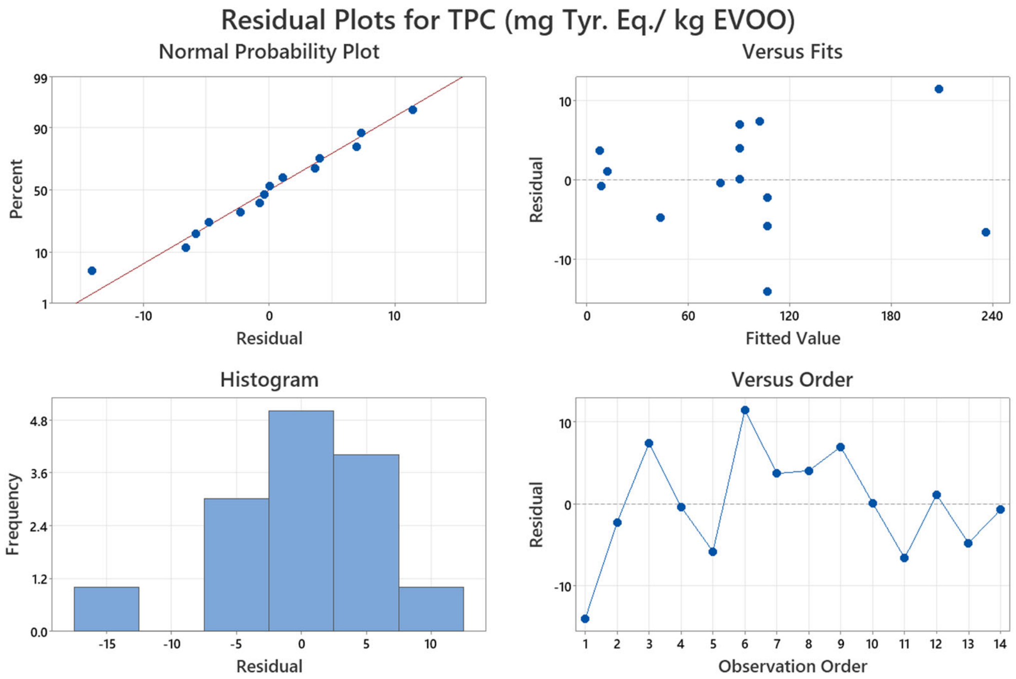

Figure S1. Residual diagnostic plots for the total phenolic content (TPC, mg Tyr. Eq./kg EVOO) second-order polynomial model fitted to the face-centered central composite design (FC-CCD). (A) Normal probability plot of residuals; (B) residuals versus fitted values; (C) histogram of residuals; and (D) residuals versus observation order (n = 14 runs).

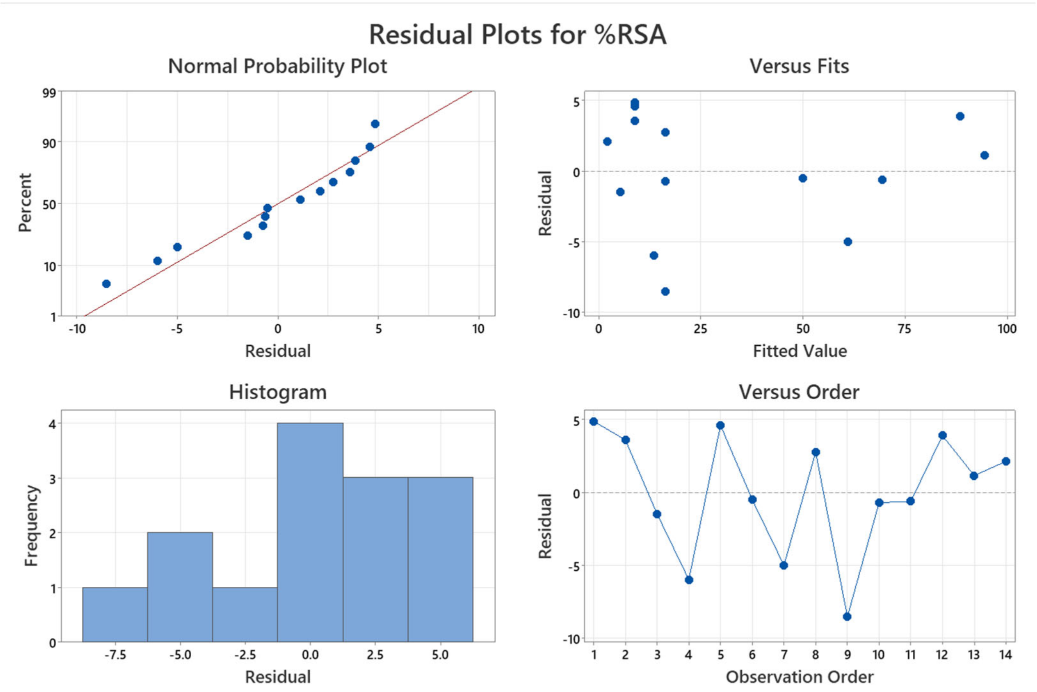

Figure S2. Residual diagnostic plots for the radical scavenging activity (%RSA, mg Tyr. Eq./kg EVOO) second-order polynomial model fitted to the face-centered central composite design (FC-CCD). (A) Normal probability plot of residuals; (B) residuals versus fitted values; (C) histogram of residuals; and (D) residuals versus observation order (n = 14 runs).

Table S2. Quality parameters of initial and residual polyphenolic EVOO following water extraction under optimized conditions.

	Acidity (%)	PV (mEq O_2 /kg)	K_{232}	K_{270}	Fruity	Bitter	Pungent
Initial	0.48± 0.02	9.62 ± 0.07	2.14±0.01	0.13 ± 0.004	6	5	4.5
Residual	0.52± 0.03	9.5±0.02	1.93±0.02	0.11±0.001	5	3	3

Acidity (% oleic acid), peroxide value (PV, expressed as mEqO 2/kg), ultraviolet light absorption values (K_{232} andK_{270}) and panel test scores, were determined according to the official methods described in EEC Reg. 2568/91 [Regulation, E. C., 2568/91. Off. J. Eur. Commun. 1991, 248,1–83].

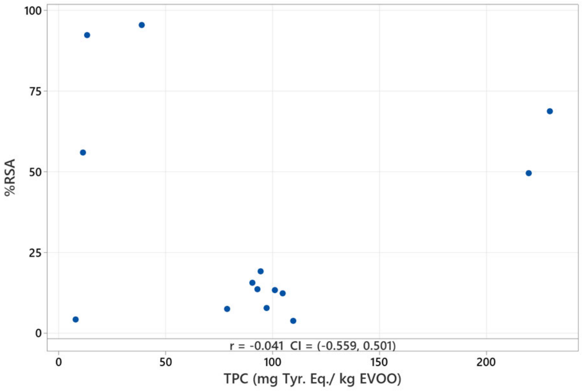

Figure S3. Correlation graph between total phenolic content (TPC, mg Tyr. Eq./kg EVOO) and radical scavenging activity (%RSA) of the aqueous EVOO extracts across the 14 runs of the face-centered central composite design (FC-CCD) (see Table 3). Pearson's correlation coefficient (r = −0.041; 95% CI: −0.559, 0.501) indicates no significant linear correlation between the two responses.

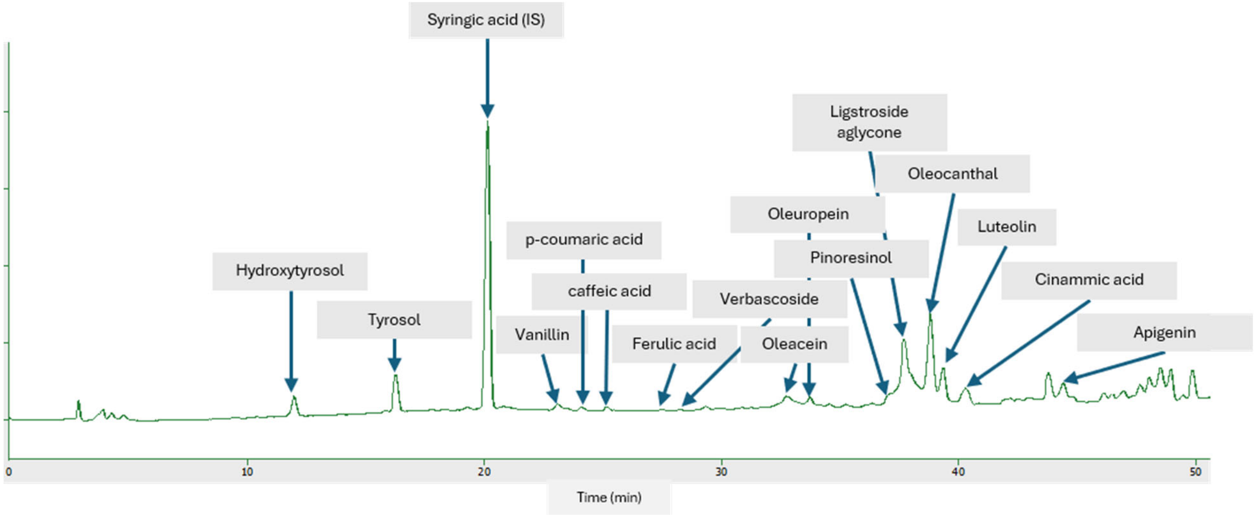

Figure S4. HPLC-DAD chromatogram of the polyphenols extracted with 80% Methanol/H2O (v/v) of EVOO, namely: Hydroxytyrosol, tyrosol, syringic acid (IS), vanillin, p-coumaric acid, caffeic acid, ferulic acid, verbascoside, oleacein, oleuropein, pinoresinol, ligstroside aglycone, oleocanthal, luteolin, cinnamic acid, apigenin).

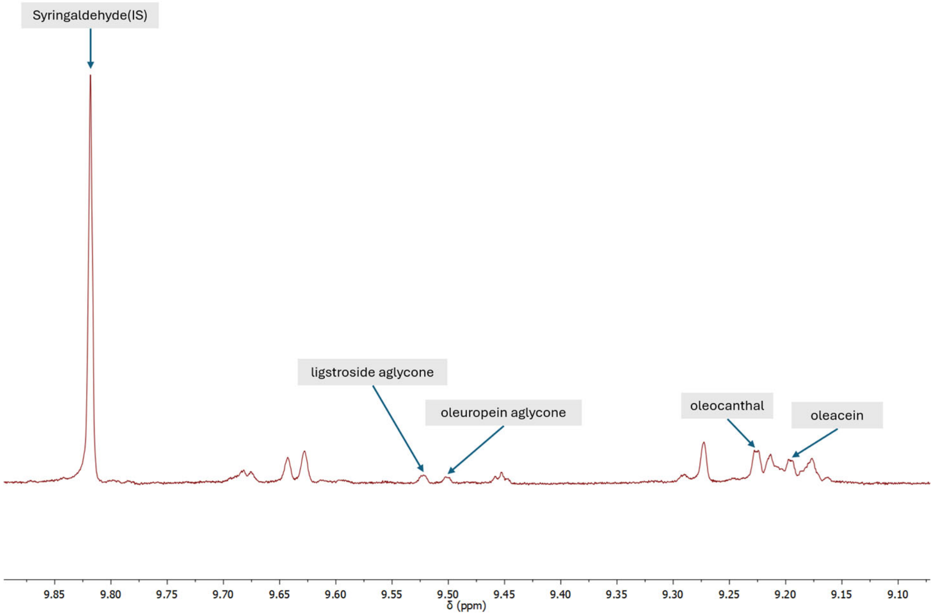

Figure S5. Aldehydic region of the ^{1}H-NMR spectrum of EVOO, showing the signals used for the quantification and integration of the internal standard syringaldehyde (9.82 ppm), ligstroside aglycone (9.52 ppm), oleuropein aglycone (9.50 ppm), oleocanthal (9.23 ppm), and oleacein (9.19 ppm).

Table S3. Single-factor experimental data for the effect of salt content on the total phenolic content (TPC, mg Tyr. Eq./kg EVOO) and radical scavenging activity (%RSA) of aqueous EVOO extracts, at a fixed water/EVOO ratio of 5. Salt content varied from 0 to 30% (w/w). Experimental values are expressed as mean ± standard deviation of triplicate measurements. Predicted values were obtained from the second-order polynomial models fitted to the FC-CCD (Table 1). The relative error (%) between experimental and predicted values was calculated as (experimental − predicted)/predicted × 100.

Salt content (%)-X_1	Ratio (Water/EVOO)-X_2	Experimental values		Predicted values		TPC-Relative error (%)	%RSA-Relative error (%)
		TPC (mg Tyr Eq./kg EVOO)	%RSA	TPC (mg Tyr Eq./kg EVOO)	%RSA		
0	5	182.1 ± 1.35	58.65 ± 0.58	188.88	55.52	3.59	5.64
5	5	150.86 ± 1.01	36.17 ± 0.12	148.64	37.25	1.49	2.90
10	5	114.84 ± 1.64	25.06 ± 0.32	116.60	24.08	1.51	4.09
15	5	91.62 ± 0.95	16.55 ± 0.07	92.73	16.00	1.20	3.46
20	5	72.96 ± 0.48	12.94 ± 0.2	77.06	13.01	5.32	0.55
25	5	67.02 ± 0.08	16.43 ± 0.74	69.57	15.12	3.67	8.65
30	5	69.47 ± 0.31	22.09 ± 0.11	70.27	22.33	1.14	1.07

Table S4. Parameters of linear regression, LOD and LOQ for phenolic compounds by HPLC analysis.

Compound	Slope	Intercept	Calibration equation	R^2	Linear range (mg L^{-1})	LOD (mg L^{-1})	LOQ (mg L^{-1})
Hydroxytyrosol	23.7721	-66.019	y = 23.7721x - 66.019	0.9994	0-500	2.283	7.179
Tyrosol	15.4143	-35.195	y = 15.4143x - 35.195	0.9996	0-500	0.196	0.616
Vanillin	36.0313	-51.289	y = 36.0313x - 51.289	0.9999	0-500	0.017	0.073
Caffeic acid	10.1825	36.571	y = 10.1825x + 36.571	1	0-250	0.059	0.182
p-Coumaric acid	20.7262	-31.266	y = 20.7262x - 31.266	0.9988	0-250	0.094	0.343
Ferulic acid	46.2605	-34.585	y = 46.2605x - 34.585	1.0000	0-250	0.017	0.064
Cinnamic acid	36.7691	4.035	y = 36.7691x + 4.035	1.0000	0-250	0.045	0.158
Verbascoside	9.7064	13.494	y = 9.7069x - 13.494	1	0-500	0.056	0.187
Oleacein	2.8897	-6.978	y = 2.8897x - 6.978	0.9996	0-1000	4.343	16.984
Oleocanthal	1.8272	0.555	y = 1.8272x + 0.555	0.9999	0-1000	2.347	8.324
Oleuropein	4.0365	-26.541	y = 4.0365x - 26.541	0.9968	0-1000	2.477	7.120
Ligstroside aglycone	13.5792	31.427	y = 13.5792x + 31.427	0.9959	0-1000	2.083	7.213

Pinoresinol	9.6908	19.921	y = 9.6908x + 19.921	1.0000	0-500	0.950	2.190
Luteolin	35.3345	-179.076	y = 35.3345x - 179.076	0.9996	0-500	0.752	2.364
Apigenin	62.8527	91.816	y = 62.8527x + 91.816	0.9974	0-500	0.025	0.092

Table S5. HPLC-DAD profile analysis of the phenolic compounds polyphenolic EVOO used for water extraction.

Sample	HPLC-DAD		
Hydroxytyrosol	15.36	±	0.729
Tyrosol	12.32	±	1.226
Vanillin	1.85	±	0.101
p-coumaric acid	2.11	±	0.029
Ferulic acid	0.642	±	0.037
Caffeic acid	0.25	±	0.003
Verbascoside	1.25	±	0.022
Oleacein	251.54	±	2.198
Oleuropein	101.98	±	0.381
Ligstroside aglycone	139.72	±	0.993
Pinoresinol	6.51	±	0.380
Oleocanthal	585.75	±	9.892
Luteolin	9.134	±	0.042
Cinnamic acid	2.42	±	0.014
Apigenin	1.478	±	0.011
Total (mg/kg)	1132.314		

Table S6. Flowability classification of powders according to their compressibility index, Hausner ratio and angle of repose values.

Flow character	Compressibility Index (%)	Hausner Ratio	Angle of Repose (°)
Excellent	1-10	1.00-1.11	25-30
Good	11-15	1.12-1.18	31-35
Fair	16-20	1.19-1.25	36-40
Passable	21-25	1.26-1.34	41-45
Poor	26-31	1.35-1.45	46-55
Very poor	32-37	1.46-1.59	56-65
Very, very poor	>38	>1.60	>66
